# Characterization of the spectra of rotating truncated gas planets and inertia–gravity modes

**DOI:** 10.1007/s00033-025-02629-0

**Published:** 2025-12-01

**Authors:** Maarten V. de Hoop, Sean Holman

**Affiliations:** 1 Computational and Applied Mathematics, and Earth Science, Rice University, Houston, TX USA; 2 Department of Mathematics, University of Manchester, Manchester, UK

**Keywords:** Essential spectrum, Inertia–gravity modes, Helmholtz decomposition, Lopatinskii conditions, 35Q85, 47A10, 35G35, 35R09

## Abstract

We study the essential spectrum, which corresponds to inertia–gravity modes, of the system of equations governing a rotating and self-gravitating gas planet. With certain boundary conditions, we rigorously and precisely characterize the essential spectrum and show how it splits from the portion of the spectrum corresponding to the acoustic modes. The fundamental mathematical tools in our analysis are a generalization of the Helmholtz decomposition and the Lopantinskii conditions.

## Introduction

We characterize the spectrum of rotating gas planets with a nonvanishing surface density, which we will call truncated, and the spectral component associated with fluid (outer) cores of rotating terrestrial planets. In the case of a polytropic model, truncation yields such a nonvanishing surface density. As commonly done, we assume the absence of viscosity of the fluid. We focus on determining the essential spectrum associated with gravito-inertial (gi) modes, in addition to the discrete spectrum associated with acoustic (p) modes, starting from the acoustic-gravitational system of linear equations for seismology supplemented with appropriate boundary conditions in a rotating reference frame. That is, in the case of gas planets we impose a vanishing (Lagrangian) pressure boundary condition. We do not impose incompressibility as the fluid does support acoustic modes. (We note that inertia–gravity modes in fluid cores of terrestrial planets are sometimes referred to as undertones [14].) While gravity modes owe their existence to a buoyancy force and inertial modes use Coriolis force as the restoring force, the inertia–gravity spectrum is controlled by both the Coriolis and buoyancy forces. For the characterization of the spectra, we introduce a modification of the classical Helmholtz decomposition and Leray projector (with range reminiscent of the anelastic approximation) while assuming “general” spatial variability in the parameters such as density of mass and Brunt–Väisälä frequency. Related work to the study of inertia–gravity modes, which we will discuss below, has taken the linearized hydrodynamics equations as a point of departure.

Assuming an incompressible fluid and homogeneity, inertial waves were studied in a rotating sphere of fluid in the laboratory [3, 27]. Kudlick [32] found an implicit solution for the eigenfrequencies of the inertial modes of a contained fluid spheroid, and Greenspan [28] calculated a pure point dense spectrum for the Poincaré’s problem (called so after Cartan [7] who followed Poincaré’s paper [38]) for cylindrical and spherical configurations. Bryan [6] was the first to give the eigenfrequencies of an incompressible axisymmetric ellipsoid including surface gravity waves. However, in the present work we do not consider the incompressible case. Ralston [41] studied the spectrum of the generator of the group of motions of an inviscid fluid in a slowly rotating container and of axisymmetric motions of a large rotating ring of fluid. He presented a family of examples exhibiting various mixtures of continuous and point spectra for this case. Colin de Verdière and Vidal [12] reproved the fact, due to Backus and Rieutord [5], that the Poincaré operator in ellipsoids admits a pure point spectrum with polynomial eigenfunctions. (Rapidly rotating fluid masses are usually ellipsoidal at the leading order [8] because of centrifugal forces and, possibly, tidal interactions due to orbital partners.) They then showed that the eigenvalues of this operator restricted to polynomial vector fields of fixed degree admit a limit repartition given by a probability measure that they construct explicitly. In this context, we mention the work by Ivers [31] on the enumeration, orthogonality, and completeness of the incompressible Coriolis modes in a triaxial ellipsoid, and the work by Maffei, Jackson, and Livermore [35] on the characterization of columnar inertial modes in rapidly rotating spheres and spheroids. A WKBJ formalism, under the assumption of a spherically symmetric structure, for inertial modes in rotating bodies and a comparison with numerical results were developed by Ivanov and Papaloizou [30].

We mention the work by Rieutord and Noui [42] studying the analogy between gravity modes and inertial modes in spherical geometry and the work by Dintrans, Rieutord, and Valdettaro [19] on gravito-inertial waves in a rotating stratified sphere or spherical shell. Vidal and Colin de Verdière [13] studied the inertia–gravity oscillations that can exist within pancake-like geophysical vortices; they considered a fluid enclosed within a triaxial ellipsoid which is stratified in density with a constant Brunt–Väisälä frequency. Results of the present work include the case considered in [13] if we replace the gravitational field of a planet by an externally imposed gravitational field on a rotating fluid.

Studies of internal oscillations and inertia–gravity modes specifically pertaining to the Earth’s fluid (outer) core, (again) with simple models, with different boundary conditions date back to the work of Olson [37] and Friedlander [23]. (Before that, Friedlander and Siegmann had studied internal oscillations in a contained rotating stratified fluid [24] and in a rotating stratified fluid in an arbitrary gravitational field [25]. Seyed-Mahmoud, Moradi, Kamruzzaman and Naseri [45] studied numerically axisymmetric compressible and stratified fluid core models with different stratification parameters in order to study the effects of the core’s density stratification on the frequencies of some of the inertia–gravity modes of this body.

WKB asymptotics of inertia–gravity modes—as well as the ray dynamics including attractors where these modes concentrate or exhibit some singularities—specifically pertaining to axisymmetric stars (but gas planets alike) without rigid boundaries were developed by Prat et al. [39, 40]. Colin de Verdière and Saint-Raymond [11] investigated spectral properties of 0th order pseudodifferential operators under natural dynamical conditions motivated by the study of (linearized) internal waves on tori. Dyatlov and Zworski [21] provided proofs of their results based on the analogy to scattering theory.

Here, we depart from assumptions invoking symmetry or homogeneity, when the component of the spectrum associated with inertia–gravity modes is no longer pure point or everywhere dense, and aim to characterize the essential spectrum in generality, starting from the full acoustic-gravitational system of (linear) equations supplemented with appropriate boundary conditions in a rotating reference frame. Indeed, the main challenge in the analysis is honoring the boundary conditions. We will not rely on any knowledge of expressions for the modes (generalized eigenfunctions), while this knowledge was essential in many of the works referenced above. In pioneering work, Valette [48, 49] presented an initial characterization of the spectrum on similar grounds. We build this, providing a mathematical description of the essential spectrum. In particular, we prove that the essential spectrum has no contributions away from the real and imaginary axes, and the portions on the axes are bounded within a certain region. Our method begins with decomposition into a component with zero dynamic pressure and a corresponding potential component. Using the Schur complement as in [47], we see that this decomposition naturally splits the spectrum into one portion that can be associated with acoustic modes, and a second component comprising intertia–gravity modes. The decomposition allows us to prove that the essential spectrum entirely corresponds with the inertia–gravity component. The inertia–gravity modes are then analyzed first using techniques from microlocal analysis due to Colin de Verdière [10] in the interior and then by reformulation into a large system of PDEs to handle the boundary. We are able to show that, when certain ellipticity conditions are satisfied, this system satisfies the Lopatinskii conditions [1], and this allows us to precisely determine the essential spectrum in Theorem 1. We also consider the bounds on the full spectrum of [22] and provide an alternate proof of those estimates, adapted to the situation we consider, in Proposition 3. Finally, we provide a partial resolution of the identity in terms of acoustic modes as this plays a role in seismological studies.

It has been noted that eigenfrequencies associated with rotational modes (originating from Liouville’s equations), which we will not consider here, are embedded in the essential spectrum [44]. It will be interesting to study viscosity limits [26], which we leave for future work. Literature already exists in this direction, including Rieutord, Valdettaro and Georgeot [43] who gave an explicit solution in the limit of vanishing viscosity for modes around attractors in a spherical shell. We mention that analysis of the essential spectrum for Maxwell’s equations with conductivity has also been carried out [2, 34] in both bounded and unbounded domains using some of the same tools.

## Acousto-gravitational system of equations and well-posedness

We consider the linearized hydrodynamics arising from perturbations of rotating self-gravitating truncated gas planets. Here the truncation is realized by setting the pressure equal to zero at the surface similar to [18]. The displacement vector of a gas or liquid parcel between the unperturbed and perturbed flow is $u^{}$. The unperturbed values of pressure (*p*), density ($\rho$), and gravitational potential ($\Phi$) are denoted with a zero subscript. We have, in a coordinate system rotating with the planet, 1 [9, 18]

1 $$\begin{aligned} \rho _0 \partial _t^2 u^{} + 2 \rho _0 \Omega \times \partial _t u^{} = \nabla ({\kappa \nabla \cdot {u^{}}}) - \nabla (\rho _0 {u^{} \cdot \nabla (\Phi _0 + \Psi ^s)}) + (\nabla \cdot (\rho _{0} u^{})) \nabla (\Phi _0 + \Psi ^s) - \rho _0 \nabla \Phi ', \end{aligned}$$

where $\kappa = p_0 \gamma$ is the bulk-modulus and $\gamma$ is the adiabatic index,

2 $$\begin{aligned} \nabla ^2 \Phi ' = -4\pi G \nabla \cdot (\rho _{0} u^{}) \end{aligned}$$

and $\Psi ^s$ denotes the centrifugal potential,

3 $$\begin{aligned} \Psi ^s = -\tfrac{1}{2} (\Omega ^2 x^2 - (\Omega \cdot x)^2) \end{aligned}$$

($|\Omega |$ signifying the rotation rate of the planet). We may introduce the solution operator, *S*, such that

4 $$\begin{aligned} \Phi ' = S(\rho _0 u^{}). \end{aligned}$$

We will use the shorthand notation,

5 $$\begin{aligned} g_0' = -\nabla (\Phi _0 + \Psi ^s). \end{aligned}$$

In fact, our results and method of proof also apply in the so-called f-plane approximation^2^ as considered in [13]. In the above, $\Omega$, $\rho _0$, $\Phi _0$ and $\kappa$ are known (unperturbed) quantities. We recognize the acoustic wave speed,

6 $$\begin{aligned} c^2 = \kappa \rho _0^{-1}. \end{aligned}$$

Typically, the underlying manifold, *M* say, is a spheroid with the axis of rotation aligned with $\Omega$. A spherically symmetric manifold requires $\Omega = 0$ from well-posedness arguments.

We rewrite the first two terms on the right-hand side of (1),

7 $$\begin{aligned} \nabla ({\kappa \nabla \cdot {u^{}}}) - \nabla (\rho _0 {u^{} \cdot \nabla (\Phi _0 + \Psi ^s)}) = \nabla [\kappa \rho _0^{-1} \, (\nabla \cdot (\rho _0 u^{}) - \tilde{s} \cdot u^{})], \end{aligned}$$

in which

8 $$\begin{aligned} \tilde{s} = \nabla \rho _0 - g_0' \frac{(\rho _0)^2}{\kappa }; \end{aligned}$$

$\tilde{s}$ is related to the Brunt–Väisälä frequency

9 $$\begin{aligned} N^2 = \rho _0^{-1} (\tilde{s} \cdot g_0'). \end{aligned}$$

We may identify $-\kappa \rho _0^{-1} \, (\nabla \cdot (\rho _0 u^{}) - \tilde{s} \cdot u^{})$ with the dynamic pressure, *P* say. (The so-called reduced pressure is given by $\rho _0^{-1} P + \Phi '$.) Thus, (1) takes the form

10 $$\begin{aligned} \partial _t^2 (\rho _0 u^{}) + 2 \Omega \times \partial _t (\rho _0 u^{}) = \nabla [c^2 \, (\nabla \cdot (\rho _0 u^{}) - \tilde{s} \cdot u^{})] - (\nabla \cdot (\rho _{0} u^{})) g_0' - \rho _0 \nabla \Phi '. \end{aligned}$$

At high eigenfrequencies, we can suppress the Coriolis force term, $2 \Omega \times \partial _t (\rho _0 u^{})$ and invoke *the Cowling approximation, when one neglects *$\nabla \Phi '$. However, we retain these terms for the moment and rewrite (10) as

11 $$\begin{aligned} \partial _t^2 (\rho _0 u^{}) + 2 \Omega \times \partial _t (\rho _0 u^{}) = \nabla [c^2 \, (\nabla \cdot (\rho _0 u^{}) - \tilde{s} \cdot u^{})] - (\nabla \cdot (\rho _0 u^{})) g_0' - \rho _0 \nabla S(\rho _0 u^{}). \end{aligned}$$

For typical models of gas giants, $\tilde{s}$ and $N^2$ are zero in a finite-thickness shell in the outer part of the planet, and we will make this assumption. In polytropic models, it can also be shown that density scales as $D^n$ near the surface of the planet, where *D* is the depth and *n* is the polytropic index. Furthermore, $c^2$ tends linearly to zero at the boundary independent of the polytropic index. Though such models, where $c^2$ and $\rho _0$ tend to zero at the boundary, are more realistic, for simplicity in this article we will apply a different boundary condition. We assume the free surface boundary condition given by the vanishing of the Lagrangian pressure perturbation, $\kappa (\nabla \cdot u)$, holds and thus get the boundary condition

12 $$\begin{aligned} [\nabla \cdot u]|_{\partial M} = 0, \end{aligned}$$

or in terms of the mass motion,

13 $$\begin{aligned} [\nabla \cdot (\rho _0 u^{}) - u^{} \cdot \nabla \rho _0]|_{\partial M} = 0. \end{aligned}$$

Analyzing the spectrum follows replacing the operator on the left-hand side of (11) by

14 $$\begin{aligned} \lambda ^2 \operatorname {Id} + 2 \lambda R_{\Omega } =: F(\lambda ),\quad R_{\Omega } \partial _t (\rho _0 u^{}) = \Omega \times \partial _t (\rho _0 u^{}), \end{aligned}$$

that is, upon replacing $\partial _t$ by $\lambda$. We introduce the shorthand notation

15 $$\begin{aligned} \rho _0 A_2 (u^{}):= -\nabla [c^2 \, (\nabla \cdot (\rho _0 u^{}) - \tilde{s} \cdot u^{})] + (\nabla \cdot (\rho _0 u^{})) g_0' + \rho _0 \nabla S(\rho _0 u^{}), \end{aligned}$$

and

16 $$\begin{aligned} L(\lambda ) = F(\lambda ) + A_2 \end{aligned}$$

identified as a quadratic operator pencil.

Let us now analyze the operator $A_2$ introduced above. We will consider the weak form of $A_2$ on the Hilbert space $H = L^2(\rho _0 \textrm{d}x)^3$. For *u* and $v \in H$ sufficiently regular, using integration by parts gives

$$\begin{aligned} \begin{aligned} \Big \langle v, A_2(u) \Big \rangle _H&= \int \limits _M \kappa (\nabla \cdot \overline{v}) (\nabla \cdot u ) + \rho _0 (\nabla \cdot \overline{v}) (g_0' \cdot u) + (g_0' \cdot \overline{v})(\nabla \cdot (\rho _0 u)) + \rho _0 \overline{v} \cdot \nabla S(\rho _0 u) \ \textrm{d} x\\&\hspace{28.45274pt}- \int \limits _{\partial M} (n \cdot \overline{v} ) \Big ( \kappa (\nabla \cdot u) + \rho _0 (g_0'\cdot u) \Big ) \ \textrm{d} s. \end{aligned} \end{aligned}$$

By the proof of [17, Lemma 4.1, Eqn (4.10)], we can rewrite the gravitational term to obtain

$$\begin{aligned} \begin{aligned} \Big \langle v, A_2(u) \Big \rangle _H&= \int \limits _M \kappa (\nabla \cdot \overline{v}) (\nabla \cdot u ) + \rho _0 (\nabla \cdot \overline{v}) (g_0' \cdot u) + (g_0' \cdot \overline{v})(\nabla \cdot (\rho _0 u)) \ \textrm{d} x\\&\hspace{28.45274pt}- \frac{1}{4 \pi G} \int \limits _{\mathbb {R}^3}\nabla S(\rho _0\overline{v}) \cdot \nabla S ( \rho _0 u) \ \textrm{d} x - \int \limits _{\partial M} (n \cdot \overline{v} ) \Big ( \kappa (\nabla \cdot u) + \rho _0 (g_0'\cdot u) \Big ) \ \textrm{d} s \end{aligned} \end{aligned}$$

If we assume boundary condition (12), we can obtain the quadratic form

17 $$\begin{aligned} \begin{aligned} a_2(v,u) = \Big \langle v, A_2(u) \Big \rangle _H&= \int \limits _M \kappa (\nabla \cdot \overline{v}) (\nabla \cdot u ) + \rho _0 (\nabla \cdot \overline{v}) (g_0' \cdot u) + (g_0' \cdot \overline{v})(\nabla \cdot (\rho _0 u)) \ \textrm{d} x\\&\hspace{28.45274pt}- \frac{1}{4 \pi G} \int \limits _{\mathbb {R}^3}\nabla S(\rho _0 \overline{v}) \cdot \nabla S (\rho _0 u) \ \textrm{d} x - \int \limits _{\partial M} (n \cdot \overline{v} ) \rho _0 (g_0'\cdot u) \ \textrm{d} s \end{aligned} \end{aligned}$$

which is symmetric because $g_0'$ and $\nabla \rho _0$ are parallel in *M* and on the boundary $\partial M$ the vectors $g_0'$ and *n* are parallel (see [17, Lemma 2.1]). Also, $a_2$ can be extended to a bounded sequilinear form on domain $H_{{{\,\textrm{Div}\,}}}(M,L^2(\partial M))$, which is the closure of $C^\infty (M) \cap H$ under the inner product

$$\begin{aligned} \langle v, u \rangle _{H_{{{\,\textrm{Div}\,}}}(M,L^2(\partial M))} = \int \limits _M \Big ( (\nabla \cdot \overline{v}) (\nabla \cdot u ) + \overline{v} \cdot u \Big ) \ \rho _0\ \textrm{d}x + \int \limits _{\partial M} (n\cdot \overline{v})(n \cdot u) \ \textrm{d} s. \end{aligned}$$

Under some hypothesis, it is then true that $a_2$ is *H*-coercive on $H_{{{\,\textrm{Div}\,}}}(M,L^2(\partial M))$. The properties mentioned above are summarized in the next lemma (see also [17] for a similar, but more complicated, case).

### Lemma 1

Suppose that *M* is compact with smooth boundary $\partial M$, *c*, $g_0' \in C(M)$, $\rho _0 \in C^1(M)$, $c^2> 0$ on *M*, $g_0'$ and $\nabla \rho _0$ are parallel in *M*, $g_0'$ and *n* are parallel on $\partial M$, and $g_0'\cdot n < 0$ on $\partial M$. Then $a_2$ defined by (17) is a continuous sequilinear form on $H_{{{\,\textrm{Div}\,}}}(M,L^2(\partial M))$. Furthermore, there exist constants $\alpha$, $\beta > 0$ such that

18 $$\begin{aligned} a_2(u,u) \ge \alpha \Vert u\Vert _{H_{{{\,\textrm{Div}\,}}}(M,L^2(\partial M))}^2 - \beta \Vert u\Vert _H^2 \end{aligned}$$

for all $$u \in H_{{{\,\textrm{Div}\,}}}(M,L^2(\partial M))$$.

### Remark 1

The hypothesis that $c^2>0$ is not realistic for gas giants as noted above since in that case $c^2$ will go to zero at $\partial M$. The requirements that the given vectors are parallel follow from hydrostatic equilibrium [17, Lemma 2.1]. Note that hydrostatic equilibrium also implies barotropic equilibrium; that is, $g_0'$, $\nabla \rho _0$ and $\nabla p_0$ are all parallel meaning level surfaces of $\rho _0$ and $p_0$ will coincide.

### Proof

Sesquilinearity follows from the hypotheses that certain vectors are parallel as can be seen from (17). Also, continuity is proven by directly applying the Cauchy–Schwartz inequality to (17). Let us now establish (18).

In the rest of the proof, *C* and *D* will always be positive constants which may change from step to step. Since *M* is compact, $c^2 \ge C > 0$ and so

$$\begin{aligned} \int \limits _M \kappa (\nabla \cdot u) (\nabla \cdot u ) \ \textrm{d} x \ge C \Vert \nabla \cdot u \Vert _H^2. \end{aligned}$$

Applying the Cauchy–Schwartz inequality and using bounds on $|g_0'|$, $\rho _0$ and $|\nabla \rho _0|$ gives, for any $\epsilon >0$

$$\begin{aligned} \left| \int \limits _M \rho _0 (\nabla \cdot u) (g_0' \cdot u) + (g_0' \cdot u)(\nabla \cdot (\rho _0 u)) \ \textrm{d} x \right| \le C \epsilon \Vert \nabla \cdot u \Vert _H^2 + D \epsilon ^{-1} \Vert u\Vert _H^2. \end{aligned}$$

Applying the definition (4) of *S*, we can bound the gravitational term

$$\begin{aligned} \left| \frac{1}{4 \pi G} \int \limits _{\mathbb {R}^3}\nabla S(\rho _0 u) \cdot \nabla S (\rho _0 u) \ \textrm{d} x \right| \le C \Vert u \Vert _H^2, \end{aligned}$$

and the fact that $g_0'$ and *n* are parallel as well as hypothesis $g_0'\cdot n < 0$ implies

$$\begin{aligned} - \int \limits _{\partial M} (n \cdot u ) \rho _0 (g_0'\cdot u) \ \textrm{d} s \ge C \Vert u\Vert ^2_{L^2(\partial M)}. \end{aligned}$$

Combining all of the previous estimates and taking $\epsilon$ sufficiently small prove (18). $\square$

Lemma 1 shows that $(H_{{{\,\textrm{Div}\,}}}(M,L^2(\partial M)),H,a_2)$ is a Hilbert triple, which implies many results about the operator $A_2$ [16, Chapter VI.3.2.5] some of which we collect in the next corollary.

### Corollary 1

The operator $A_2$ is continuous from $H_{{{\,\textrm{Div}\,}}}(M,L^2(\partial M))$ to the Hilbert dual $H_{{{\,\textrm{Div}\,}}}(M,L^2(\partial M))'$. Also, $A_2$ is an unbounded self-adjoint operator on *H* with domain

$$\begin{aligned} D(A_2) = \{ u \in H_{{{\,\textrm{Div}\,}}}(M,L^2(\partial M)) \, \ v \mapsto a_2(u,v) \text{ is } \text{ continuous } \text{ with } \text{ respect } \text{ to } \text{ the } L^2(M,\rho _0 \ \textrm{d} x) \text{ norm }\}. \end{aligned}$$

By analyzing (17) and the equation before, we can say more about $D(A_2)$ if we make additional regularity assumptions about the parameters. This is done in the next corollary.

### Corollary 2

In addition to the hypotheses of Lemma 1, assume that $c^2 \in C^1(M)$ and $\rho _0 \in C^2(M)$. Then

$$\begin{aligned} D(A_2) = \{ u \in H_{{{\,\textrm{Div}\,}}}(M,L^2(\partial M)) \, | \, \nabla [c^2 \, (\nabla \cdot (\rho _0 u^{}) - \tilde{s} \cdot u^{})] \in L^2(M), \ [\nabla \cdot u]|_{\partial M} = 0 \}. \end{aligned}$$

## Decompositions of Hilbert space and spectrum

Our main goal is to characterize the spectrum of the operator pencil $L(\lambda )$ given by (16). To this end, let us recall the definition of the resolvent set, spectrum and essential spectrum as given, for example, in [36].

### Definition 1

Let $\Omega \subset \mathbb {C}$ be an open set and for each $\lambda \in \Omega$ suppose $T(\lambda )$ is a closed linear operator from $D(T(\lambda )) \subset H$ to *H*. The set of $\lambda \in \Omega$ such that $T(\lambda )$ is bijective on its domain with bounded inverse $T(\lambda )^{-1}:H\rightarrow H$ is the resolvent set of *T* which we will notate as $\rho (T)$. The complement of the resolvent set is the spectrum $\sigma (T)$, and the set of $\lambda \in \Omega$ such that $T(\lambda )$ is not a Fredholm operator^3^ is the essential spectrum $\sigma _{ess}(T)$.

Note that by Lemma 1 and the Lax–Milgram Theorem (see, for example, [16, Theorem 7, p 368]), for $\textrm{Re}\ \lambda ^2 > \beta$ we know that $L(\lambda )$ has bounded inverse and so such $\lambda$ are in the resolvent set $\rho (L)$. However, as we will see below after considering an appropriate decomposition of *H*, $L(\lambda )^{-1}$ is not compact meaning that the analytic Fredholm theory cannot be applied as in, for example, [36, Lemma 1.2.1, p 7] and the essential spectrum $\sigma _{ess}(L)$ is not empty.

We now develop an orthogonal decomposition generalizing the Helmholtz decomposition,

19 $$\begin{aligned} H = H_1 \oplus H_2, \end{aligned}$$

with corresponding projections,

$$\begin{aligned} \pi _{1,2}:\ H \rightarrow H_{1,2}, \end{aligned}$$

with the goal to extract the part of the point spectrum associated with the acoustic normal modes and characterize the essential spectrum. The construction of the decomposition will entail the introduction of a space $E_1$ such that the injection of $E_1$ into $H_1$ is compact.

We introduce the operator

20 $$\begin{aligned} T u:= \nabla \cdot (\rho _0 u) - \tilde{s} \cdot u = \rho _0 [\nabla \cdot u + \rho _0 \kappa ^{-1} g_0' \cdot u],\quad D(T) = H_{{{\,\textrm{Div}\,}},0}(M) = \{ u \in H_{{{\,\textrm{Div}\,}}}(M) \, \ u \cdot n|_{\partial M} = 0\}, \end{aligned}$$

which sets the dynamic pressure to zero (and induces the so-called *anelastic* approximation). The adjoint, $T^*$, of *T* is given by

21 $$\begin{aligned} T^* \varphi = -(\nabla (\rho _0 \varphi ) + \tilde{s} \varphi ) \end{aligned}$$

with $D(T^*) = H^1(M)$. Operators *T*, $T^*$, have the following properties.

### Lemma 2

Assume the hypotheses of Lemma 1. Then $\operatorname {Ran}(T^*)$ is closed in *H* and $H = \operatorname {Ran}(T^*)\oplus \operatorname {Ker}(T)$. Moreover, the map $\Pi : H \rightarrow H^1(M)$ taking *u* to the unique minimum norm $\varphi \in H^1(M)$ satisfying $u = T^* \varphi + u_2$ for $u_2 \in \operatorname {Ker}(T)$ is continuous. Finally, the injection $\operatorname {Ran}(T^*) \, \cap \, D(T) \hookrightarrow H$, with the $H_{{{\,\textrm{Div}\,}}}(M)$ topology on the domain, is compact.

### Proof

The decomposition $H =\overline{\operatorname {Ran}(T^*)} \oplus \operatorname {Ker}(T)$ is a general fact for a closed operator *T* with dense domain. In this case, $D(T) = H_{{{\,\textrm{Div}\,}},0}(M)$ is dense in *H* as in [48, Prop. 2, p. 67] it is stated that $C_0^\infty \subset H_{{{\,\textrm{Div}\,}},0}(M)$ is dense in $H_{{{\,\textrm{Div}\,}}}(M)$ and then $\overline{H_{{{\,\textrm{Div}\,}}}(M)}=H$. The next part of the proof follows the method of [46, Chap]. To show that $\operatorname {Ran}(T^*)$ is closed, consider the operator $\mathcal {L}_{T^*}: H^1(M) \rightarrow H^1(M)^*$ defined by

$$\begin{aligned} \langle \mathcal {L}_{T^*} \varphi ,\psi \rangle = \langle T^* \varphi , T^* \psi \rangle _H \quad \forall \varphi , \ \psi \in H^1(M). \end{aligned}$$

It is possible to find a real constant *C* sufficiently large so that

$$\begin{aligned} \langle (\mathcal {L}_{T^*}+C) \varphi ,\varphi \rangle = \langle T^* \varphi , T^* \varphi \rangle _H + C \langle \varphi ,\varphi \rangle _{L^2(\rho _0 \ \textrm{d} x)} \ge \tilde{C} \Vert \varphi \Vert _{H^1(M)}^2 \quad \forall \varphi \in H^1(M). \end{aligned}$$

Following the method of [46], we deduce that $\mathcal {L}_{T^*} + C$ is one to one and onto, and by considering the inverse of $\mathcal {L}_{T^*} + C$, which is a compact and self-adjoint operator, we find that there exists an orthonormal basis of eigenvectors on *H* for $\mathcal {L}_{T^*}$ with discrete eigenvalues going to infinity. Since $\mathcal {L}_{T^*}$ is a non-negative operator by its definition, the corresponding eigenvalues must all be non-negative with the possibility that zero is an eigenvalue with finite multiplicity. Thus, the kernel of $\mathcal {L}_{T^*}$ is finite dimensional, and note that it also coincides with kernel of $T^*$.

Now, suppose that $\{ y_n \}_{n=1}^\infty \subset \operatorname {Ran}(T^*)$ is a Cauchy sequence for *H*. Then there exists a sequence $\{\varphi _n \}_{n=1}^\infty \subset H^1(M)$ such that $y_n = T^* \varphi _n$ and without loss of generality we can assume that all $\varphi _n$ are orthogonal to the kernel of $\mathcal {L}_{T^*}$. By taking the smallest positive eigenvalue of $\mathcal {L}_{T^*}$, which is strictly greater than zero by the above argument, we therefore show that $\varphi _n$ is a Cauchy sequence in $L^2(\rho _0 \ \textrm{d} x)$. From the formula (21) of $T^*$, as well as facts that $\rho _0 \in C^1(M)$ is positive and $\tilde{s} \in C(M)^3$, we have the inequality

$$\begin{aligned} \Vert y_n - y_m\Vert ^2_{H} + C \Vert \varphi _n - \varphi _m\Vert ^2_{L^2(\rho _0 \ \textrm{d} x)} \ge C \Vert \nabla (\varphi _n-\varphi _m) \Vert ^2_{H} \end{aligned}$$

for some $C>0$ possibly different from the one above. Therefore, $\{\varphi _n\}_{n=1}^\infty$ is a Cauchy sequence in $H^1(M)$ and since $H^1(M)$ is complete it must converge. This implies that $\{y_n\}_{n=1}^\infty$ must also converge in *H* to a point in $\operatorname {Ran}(T^*)$. Since *H* is a complete space, this proves that $\operatorname {Ran}(T^*)$ is closed.

Using its eigendecomposition, we can find a pseudoinverse for $\mathcal {L}_{T^*}$ which we will denote $\mathcal {L}_{T^*}^{\dagger }: H^1(M)^* \rightarrow H^1(M)$. To prove the continuity of the map $\Pi$, note that, after extending *T* to an operator from *H* to $H^1(M)^*$ by duality,

$$\begin{aligned} \Pi = \mathcal {L}_{T^*}^\dagger T. \end{aligned}$$

That the injection, $$\operatorname {Ran}(T^*) \, \cap \, D(T) \hookrightarrow H$$, is compact follows exactly as in [48, Prop 3d, p. 70] where it was done for the case $$g_0'=0 $$ using the Rellich–Kondrashov compactness theorem for Sobolev spaces. The proof is straightforwardly adapted to $$g_0'\ne 0.$$ $$\square $$

Given Lemma 2, we obtain the decomposition (19) by setting $H_1 = \operatorname {Ran}(T^*)$ and $H_2 = \operatorname {Ker}(T)$. This is a generalization of the Helmholtz decomposition which is required for our analysis. Using the proof of Lemma 2, the projection onto $\operatorname {Ran}(T^*)$, generalizing the notion of irrotational, is given by $T^* \mathcal {L}_{T^*}^\dagger T$ and the projection onto $\operatorname {Ker}(T)$, generalizing the notion of anelastic, is given by $I - T^* \mathcal {L}_{T^*}^\dagger T$. These formulae lead to the next lemma.

### Lemma 3

The orthogonal projection operators $\pi _1: H \rightarrow \textrm{Ran}(T^*) = H_1$ and $\pi _2: H \rightarrow \textrm{Ker}(T) = H_2$ are zero-order pseudodifferential operators in the interior of *M* with principal symbols given, respectively, by

22 $$\begin{aligned} \sigma _p(\pi _1) = \frac{\xi \xi ^T}{|\xi |^2}, \quad \sigma _p(\pi _2) = \textrm{Id} - \frac{\xi \xi ^T}{|\xi |^2}. \end{aligned}$$

### Proof

For $u \in H$, suppose that $u_1 = T^* \Pi (u) = \pi _1(u)$ and $u_2 = \pi _2(u)$. Thus

$$\begin{aligned} u = T^* \Pi (u) + u_2 \Rightarrow Tu = TT^* \Pi (u). \end{aligned}$$

$TT^*$ is an elliptic second-order differential operator and as such has a pseudodifferential parametrix on the interior of *M*, which is an order $-2$ pseudodifferential operator $(TT^*)^{-1}$ there such that

23 $$\begin{aligned} (TT^*)^{-1} T u = \Pi (u) + Ku \end{aligned}$$

where *K* is a smoothing operator in the interior of *M*. Therefore,

$$\begin{aligned} \pi _1(u) = T^* (TT^*)^{-1} T u - T^* Ku. \end{aligned}$$

This proves that $\pi _1$ is a zero-order pseudodifferential operator in the interior of *M*. By looking at the prinicpal symbols of *T* and $T^*$ and using the composition calculus, we conclude that $\sigma _p(\pi _1)$ is as given in (22). Since $\pi _2 = \textrm{Id} - \pi _1$, the conclusion for $\sigma _p(\pi _2)$ follows as well. $\square$

Our next task is to decompose the operator $A_2$. Toward this end, we introduce

$$\begin{aligned} E_1 = D(A_2) \cap \operatorname {Ran}(T^*) \end{aligned}$$

and

$$\begin{aligned} E_2 = D(A_2) \cap \operatorname {Ker}(T), \end{aligned}$$

whence

$$\begin{aligned} E_1 \oplus E_2\subset D(A_2). \end{aligned}$$

For the opposite inclusion, consider $u \in D(A_2)$. Since $\pi _2(u) \in \operatorname {Ker}(T)$, we see that $\nabla c^2Tu = 0$ and, since *n* is parallel with $g_0'$ on $\partial M$,

$$\begin{aligned} 0 = \rho _0 \kappa ^{-1} g_0' \cdot u |_{\partial M} = \nabla \cdot u|_{\partial M}. \end{aligned}$$

Therefore, referring to Corollary 2, $\pi _2(u) \in D(A_2)$ and so

$$\begin{aligned} E_1 \oplus E_2= D(A_2). \end{aligned}$$

We now aim to introduce a corresponding block decomposition of the operator $L(\lambda )$ introduced in (16). Indeed, let us define the component operators by

$$\begin{aligned} L_{ij}(\lambda ) = \pi _i L(\lambda ) \pi ^*_j \end{aligned}$$

for *i*, $j = 1$ and 2. Considering $D(A_2)$ in Corollary 2 and noting that $\operatorname {Ker}(T) \subset D(A_2)$, we see that

$$\begin{aligned} D(L_{i1}(\lambda )) = E_1, \quad D(L_{i2}(\lambda )) = \operatorname {Ker}(T) \end{aligned}$$

for $i = 1$ and 2. With these operators, we see that $L(\lambda )$ is related to these component operators by

$$\begin{aligned} L(\lambda ) = \begin{pmatrix} \pi _1^*&\pi _2^* \end{pmatrix} \begin{pmatrix} L_{11}(\lambda ) &  L_{12}(\lambda ) \\ L_{21}(\lambda ) &  L_{22}(\lambda ) \end{pmatrix} \begin{pmatrix} \pi _1 \\ \pi _2 \end{pmatrix} \end{aligned}$$

and thus, the resolvent set, spectrum, and essential spectrum of $L(\lambda )$ are equivalent to the same for the block matrix on the right side of the equation which we label as

$$\begin{aligned} \mathcal {L}(\lambda ) = \begin{pmatrix} L_{11}(\lambda ) &  L_{12}(\lambda ) \\ L_{21}(\lambda ) &  L_{22}(\lambda ) \end{pmatrix}, \quad D(\mathcal {L}(\lambda )) = E_1 \oplus \operatorname {Ker}(T). \end{aligned}$$

In the next proposition, we summarize some of the properties of the component operators.

### Proposition 1

Suppose that $g_0' \in C(M)$ and $\rho _0 \in C^1(M)$. Then the operators $L_{12}(\lambda ): \operatorname {Ker}(T) \rightarrow \operatorname {Ran}(T^*)$ and $L_{22}(\lambda ): \operatorname {Ker}(T) \rightarrow \operatorname {Ker}(T)$ are bounded. The operator $L_{21}(\lambda )$ with domain $E_1$ is closable with closure a bounded operator from $\operatorname {Ran}(T^*)$ to $\operatorname {Ker}(T)$. Finally, $L_{11}(\lambda )$ with domain $E_1$ is a Fredholm operator with index 0 and discrete spectrum consisting of eigenvalues which have finite multiplicity. Further, $L_{11}(\lambda )^{-1}$ is compact on the resolvent set of $L_{11}$.

### Proof

Suppose that $u \in \operatorname {Ker}(T)$. Then from (15), (16) and (20),

24 $$\begin{aligned} L(\lambda ) u = F(\lambda ) u + \frac{\tilde{s} \cdot u}{\rho _0} g_0' + \nabla S(\rho _0 u). \end{aligned}$$

The first two terms on the right side above are clearly bounded as they are only multiplication by bounded quantities. The third term, corresponding to self-gravitation is also bounded by Lemma 4 which is proved below. Because the projectors $\pi _1$ and $\pi _2$ are both continuous this proves the boundedness of $L_{12}(\lambda )$ and $L_{22}(\lambda )$ as stated.

Now let us consider $L_{21}(\lambda )$. Taking $u \in E_1$ and $v \in \operatorname {Ker}(T)$ we have

$$\begin{aligned} \begin{aligned} \langle A_2 u, v \rangle _H&= \langle u, A_2 v \rangle _H \\&= \left\langle u, \frac{\tilde{s} \cdot v}{\rho _0} g_0' + \nabla S(\rho _0 v) \right\rangle _H\\&= \left\langle \frac{g_0' \cdot u}{\rho _0} \tilde{s} + \nabla S(\rho _0 u), v \right\rangle _H. \end{aligned} \end{aligned}$$

Since this is true for any $v \in \operatorname {Ker}(T)$, we conclude that

$$\begin{aligned} L_{21}(\lambda ) u = \pi _2 \left( F(\lambda ) u + \frac{g_0' \cdot u}{\rho _0} \tilde{s} + \nabla S(\rho _0 u)\right) . \end{aligned}$$

Similar to above, this is a bounded operator and so $L_{21}(\lambda )$ extends to a bounded operator from $\operatorname {Ran}(T^*)$ to $\operatorname {Ker}(T)$ as claimed.

Finally, the statement about $L_{11}(\lambda )$ follows by the argument of [36, Lemma 1.1.11] and the compactness of resolvent of $\pi _1 A_2 \pi _1^*$ which is a consequece of Lemma 2. Indeed, since $E_1 \subset H_{{{\,\textrm{Div}\,}}}(M,L^2(\partial M))$ by Lemma 1$\pi _1 A_2 \pi _1^* + \beta I$ is invertible from its domain $E_1$ into $\operatorname {Ran}(T^*)$ and, by the closed graph theorem, the inverse is bounded with the graph norm on $E_1$. Since the injection $E_1 \hookrightarrow \operatorname {Ran}(T^*) \cap D(T)$, with the graph norm on $E_1$ and $H_{{{\,\textrm{Div}\,}}}(M)$ topology on $\operatorname {Ran}(T^*) \cap D(T)$ is continuous, by Lemma 2$(\pi _1 A_2 \phi _1^* + \beta I)^{-1}: \operatorname {Ran}(T^*) \rightarrow \operatorname {Ran}(T^*)$ is compact. Furthermore, we have the identity

$$\begin{aligned} \pi _1 A_2 \pi _1^* (\pi _1 A_2 \pi _1^* + \beta I)^{-1} = I - \beta (\pi _1 A_2 \pi _1^* + \beta I)^{-1} \end{aligned}$$

which when applied to $L_{11}(\lambda )$ gives

25 $$\begin{aligned} L_{11}(\lambda ) (\pi _1 A_2 \pi _1^* + \beta I)^{-1} = I + (\pi _1 F(\lambda ) \pi _1^* - \beta I) (\pi _1 A_2 \pi _1^* + \beta I)^{-1}. \end{aligned}$$

This operator is therefore a compact pertubation of the identity and so $L_{11}(\lambda )$ is Fredholm with index 0 as claimed in the statement of the proposition. The fact that $L_{11}(\lambda )$ has discrete spectrum consisting of eigenvalues with finite multiplicity then follows from the analytic Fredholm theory. For $\lambda$ in the resolvent set of $L_{11}$, we can apply $L_{11}^{-1}$ to (25) and get

$$\begin{aligned} L_{11}^{-1}(\lambda ) = ((1-\beta )I - \pi _1 F(\lambda ) \pi _1^*) (\pi _1 A_2 \pi _1^* + \beta I)^{-1} \end{aligned}$$

which is compact. $\square$

### Remark 2

If we additionally assume that $g_0'$ and $\nabla \rho _0$ are parallel, which is a requirement for well-posedness of the system (1), and use the Brunt–Väisälä frequency $N^2$ (see (9)), the proof of Proposition 1 implies the following formulae

26 $$\begin{aligned} \begin{aligned} L_{12}(\lambda )&= \pi _1 \left( F(\lambda ) + N^2 \hat{g}_0' \hat{g}_0'^T + \nabla S \rho _0 \right) \pi _2^*, \quad L_{22}(\lambda ) = \pi _2 \left( F(\lambda ) + N^2 \hat{g}_0' \hat{g}_0'^T + \nabla S \rho _0 \right) \pi _2^*,\\ L_{21}(\lambda )&= \pi _2 \left( F(\lambda ) + N^2 \hat{g}_0' \hat{g}_0'^T + \nabla S \rho _0 \right) \pi _1^*, \end{aligned} \end{aligned}$$

where

$$\begin{aligned} \hat{g}_0' = \frac{g_0'}{\Vert g_0'\Vert }. \end{aligned}$$

From these formulae and Lemma 4, $L_{22}(\lambda )$ cannot have a compact inverse. Thus by taking $u \in \operatorname {Ker}(T)$ we see that $L(\lambda )^{-1}$ cannot be compact as observed earlier.

Before continuing, we prove a technical lemma which was used in the proof of Proposition 1 and will also be important below.

### Lemma 4

The map $u \rightarrow S(\rho _0 u)$ is continuous from $L^2(M)$ to $L^6(\mathbb {R}^3)$ and the map $u \rightarrow \nabla S(\rho _0 u)$ is continuous from $L^2(M)$ to $L^2(\mathbb {R}^3)$. The map $u \rightarrow \nabla S(\rho _0 u)$ is compact from $H_{{{\,\textrm{Div}\,}}}(M,L^2(\partial M))$ to $L^2(\mathbb {R}^3)$.

### Proof

Starting from the definition given by (2) and (4) of *S*, setting $v = \rho _0 u$, and using Parseval’s identity, we have

27 $$\begin{aligned} \begin{aligned} \Vert \nabla S(v) \Vert ^2_{L^2(\mathbb {R}^3)^3} = (4 \pi G)^2 \int \limits _{\mathbb {R}^3} \frac{|\hat{v}(\xi ) \cdot \xi |^2}{|\xi |^{2}} \textrm{d}\xi \le (4 \pi G)^2 \int \limits _{\mathbb {R}^3} |\hat{v}(\xi )|^2 \textrm{d}\xi = (4 \pi G)^2 \Vert v \Vert _{L^2(M)^3}^2. \end{aligned} \end{aligned}$$

From (27), we conclude that

(i) $u \mapsto \nabla S(\rho _0 u)$ is a bounded operator from $L^2(M)^3$ to $L^2(\mathbb {R}^3)^3$, and accordingly that
(ii) $u \mapsto S(\rho _0 u)$ is a bounded operator from $L^2(M)^3$ into $L^6(\mathbb {R}^3)^3$ using the Gagliardo–Nirenberg–Sobolev inequality.

Thus, the first two assertions of the lemma have been proved.

Regarding the third assertion, we use (2) together with Parseval’s identity in writing

28 $$\begin{aligned} \Vert \nabla S(v) \Vert ^2_{L^2(\mathbb {R}^3)^3} = 4 \pi G \langle \nabla S(v), v \rangle _{L^2(\mathbb {R})^3} = 4 \pi G \langle \nabla S(v), v \rangle _{L^2(M)^3}. \end{aligned}$$

For $v\in H_{{{\,\textrm{Div}\,}}}(M,L^2(\partial M))$, integration by parts gives

29 $$\begin{aligned} \langle \nabla S(v), v \rangle _{L^2(M)^3} = \langle S(v), n\cdot v \rangle _{L^2(\partial M)} - \langle S(v), \nabla \cdot v \rangle _{L^2(M)^3}. \end{aligned}$$

Now consider any bounded sequence $\{ u^\ell \} \subset H_{{{\,\textrm{Div}\,}}}(M,L^2(\partial M))$. By Alaoglu’s Theorem, there is a subsequence that converges weakly in $H_{{{\,\textrm{Div}\,}}}(M,L^2(\partial M))$ to a point $\widetilde{v} \in H_{{{\,\textrm{Div}\,}}}(M,L^2(\partial M))$. Then the subsequence $\{v^{\ell _k} - \widetilde{v} \}$ is bounded in $H_{{{\,\textrm{Div}\,}}}(M,L^2(\partial M))$ and converges weakly to zero. Now, note that by (i) and (ii), and using also Hölder’s inequality, $S:L^2(M)^3 \rightarrow H^1(M)^3$ is continuous and so $S:L^2(M)^3 \rightarrow L^2(M)^3$ is compact as well as the composition of *S* with restriction to the boundary. Thus, by taking a further subsequence if necessary, $\{S(v^{\ell _k} - \widetilde{v})\}$ converges strongly to some point $w \in L^2(M)^3$. Combining (28) and (29) and applying to $v^{\ell _k} - \widetilde{v}$, we have

$$\begin{aligned} \Vert \nabla S (v^{\ell _k}) - \nabla S (\widetilde{v})\Vert ^2_{L^2(\mathbb {R}^3)^3} = \langle S(v^{\ell _k} - \widetilde{v}), n\cdot (v^{\ell _k} - \widetilde{v})\rangle _{L^2(\partial M)} - \langle S(v^{\ell _k} - \widetilde{v}), \nabla \cdot (v^{\ell _k} - \widetilde{v})\rangle _{L^2(M)^3}. \end{aligned}$$

Since $S(v^{\ell _k} - \widetilde{v})$ converges strongly to *w*, $v^{\ell _k} - \widetilde{v}$ is bounded, and $v^{\ell _k} - \widetilde{v}$ converges weakly to zero, we obtain that

$$\begin{aligned} \lim _{k\rightarrow \infty } \Vert \nabla S (v^{\ell _k}) - \nabla S (\widetilde{v})\Vert _{L^2(\mathbb {R}^3)^3} = 0. \end{aligned}$$

This completes the proof. $\square$

We now apply Frobenius–Schur factorization to the operator $\mathcal {L}(\lambda )$ to draw conclusions about the decomposition of its spectrum. Our factorization and resulting spectral decomposition are essentially the same as [47, Theorem 2.2.14], except that we consider quadratic dependence on $\lambda$. This does not introduce any serious complication into the method. Suppose that $\rho _1$ is the resolvent set of $L_{11}$ and $\rho _2$ the resolvent set of $L_{22}$ with complements $\sigma _1$ and $\sigma _2$ the corresponding spectra. For $\lambda \in \rho _1$, we define the Schur complement

$$\begin{aligned} S_2(\lambda ) = L_{22}(\lambda ) - L_{21}(\lambda ) L_{11}(\lambda )^{-1} L_{12}(\lambda ) \end{aligned}$$

and similarly for $\lambda \in \rho _2$ we have

$$\begin{aligned} S_{1}(\lambda ) = L_{11}(\lambda ) - L_{12}(\lambda ) L_{22}(\lambda )^{-1} L_{21}(\lambda ). \end{aligned}$$

Then for $$\lambda \in \rho _1$$

$$\begin{aligned} \mathcal {L}(\lambda ) = \begin{pmatrix} I &  0 \\ L_{21}(\lambda ) L_{11}^{-1}(\lambda ) &  I \end{pmatrix} \begin{pmatrix} L_{11}(\lambda ) &  0 \\ 0 &  S_2(\lambda ) \end{pmatrix} \begin{pmatrix} I &  L_{11}^{-1}(\lambda ) L_{12}(\lambda ) \\ 0 &  I \end{pmatrix}, \end{aligned}$$

while for $$\lambda \in \rho _2$$

30 $$\begin{aligned} \mathcal {L}(\lambda ) = \begin{pmatrix} I &  L_{12}(\lambda ) L_{22}^{-1}(\lambda ) \\ 0 &  I \end{pmatrix} \begin{pmatrix} S_1(\lambda ) &  0 \\ 0 &  L_{22}(\lambda ) \end{pmatrix} \begin{pmatrix} I &  0 \\ L_{22}^{-1}(\lambda ) L_{21}(\lambda ) &  I \end{pmatrix}. \end{aligned}$$

By [47, Lemma 2.3.2], since the matrix operators on the outside of the products in each equality above are invertible on the respective resolvent sets, we obtain

$$\begin{aligned} \sigma (L) \setminus \sigma _1 = \sigma (S_2), \quad \sigma (L) \setminus \sigma _2 = \sigma (S_1). \end{aligned}$$

The same statement for essential spectrum is not explicitly given by [47, Lemma 2.3.2] but follows by the same proof. This means

$$\begin{aligned} \sigma _{ess}(L) \setminus \sigma _{ess}(L_{11}) = \sigma _{ess}(S_2), \quad \sigma _{ess}(L) \setminus \sigma _{ess}(L_{22}) = \sigma _{ess}(S_1). \end{aligned}$$

By Proposition 1, $\sigma _{ess}(L_{11}) = \emptyset$ and so in fact we have

$$\begin{aligned} \sigma _{ess}(L) = \sigma _{ess}(S_2). \end{aligned}$$

Using identity (25), we have

$$\begin{aligned} S_1(\lambda ) (\pi _1 A_2 \pi _1^* + \beta I)^{-1} = I + (\pi _1 F(\lambda ) \pi _1^* - \beta I + L_{12}(\lambda ) L_{22}^{-1}(\lambda ) L_{21}(\lambda )) (\pi _1 A_2 \pi _1^* + \beta I)^{-1} \end{aligned}$$

and so since $L_{12}(\lambda ) L_{22}^{-1}(\lambda ) L_{21}(\lambda )$ is bounded for $\lambda \in \rho _2$ we obtain that, as in (25), $S_1(\lambda )$ is Fredholm with index 0 having only eigenvalues with finite multiplicity in its spectrum. We summarize the main results just proved in the following proposition.

### Proposition 2

The spectrum of $\sigma (S_1)$ is discrete:

$$\begin{aligned} \sigma (S_1) \subseteq \sigma _{\textrm{disc}}(L), \end{aligned}$$

where $\sigma _{\textrm{disc}}(L)$ denotes the discrete component of $\sigma (L)$. Furthermore,

$$\begin{aligned} \sigma _{ess}(L) = \sigma _{ess}(S_2). \end{aligned}$$

### Remark 3

There are eigenvalues of *L* that do not lie in $\sigma _1$. Specifically, the quasi-rigid modes form three separate two-dimensional eigenspaces with eigenvalues $\pm \textrm{i}|\Omega |$ and 0. These eigenvalues are embedded in the essential spectrum. For the sake of self-containedness, the detailed computations are given in Appendix A.

### Remark 4

(Geostrophic modes) For completeness of the characterization, we briefly present how the geostropic modes (see [15, Section 4.1.6]) appear in the analysis. Fluid motions which travel along the level surfaces of $\rho _0$ and preserve the density are eigenfunctions of *L*, or geostrophic modes, corresponding to $\lambda = 0$.

They are necessarily solutions to the problem

31 $$\begin{aligned} \left\{ \begin{array}{ll} & \tilde{s}\cdot u=0,\\ & \nabla \cdot (\rho _0 u)=0,\\ & \nabla \cdot u |_{\partial M}=0. \end{array} \right. \end{aligned}$$

Note that if $u \in H$ satisfies (31), then $u \in H_2 = \operatorname {Ker}(T)$. If $\varphi \in H^1(M)$ is such that

32 $$\begin{aligned} \nabla \varphi \cdot (\nabla \times \tilde{s})=0 \end{aligned}$$

and we define *u* by

33 $$\begin{aligned} u=\rho _0^{-1}\nabla \varphi \times \tilde{s}, \end{aligned}$$

then *u* satisfies the first and the second equations of (31) as $\nabla \cdot (\nabla \varphi \times \tilde{s})=\tilde{s}\cdot (\nabla \times \nabla \varphi )-\nabla \varphi \cdot (\nabla \times \tilde{s}).$ Since we have also

$$\begin{aligned} \nabla \cdot (\rho _0^{-1}\nabla \varphi \times \tilde{s})=(\nabla \varphi \times \tilde{s})\cdot \nabla \rho _0^{-1}, \end{aligned}$$

the boundary condition in (31) is equivalent to

$$\begin{aligned} (\nabla \varphi \times \tilde{s})\cdot \nabla \rho _0^{-1} |_{\partial M}= 0. \end{aligned}$$

Assuming that $g_0'$, $\nabla \rho _0$ and *n* are parallel on $\partial M$, which is required for well-posedness of the system, this boundary condition is automatically satisfied. In conclusion, the geostrophic modes form a infinite-dimensional subspace of $H_2.$ This is consistent with the fact that the essential spectrum of *L* corresponds with the $H_2$ component (i.e., *L*(0) fails to be Fredholm because of an infinite-dimensional kernel contained in $H_2$).

## Riesz projectors and acoustic mode decomposition

A common approach to solution of (1) is to expand *u* in so-called acoustic modes. In practice, this typically means expansion in the eigenfunctions of $L_{11}$, which by Proposition 1 correspond to discrete eigenvalues. Indeed, applying the spectral theory on Krein spaces ( [4, 33]) and properties from Proposition 1 it is possible to develop a resolution of the identity for $L_{11}$ using its eigenfunctions. However, these eigenfunctions are not modes for the operator *L*. Indeed, suppose $\lambda \in \rho _2$, which by Proposition 2 is outside of the essential spectrum of *L*. Using the decomposition (30), we have that

$$\begin{aligned} \mathcal {L}(\lambda ) \begin{pmatrix} u \\ v \end{pmatrix} = 0 \end{aligned}$$

if and only if

$$\begin{aligned} S_1(\lambda ) u = 0, \quad L_{22}^{-1}(\lambda ) L_{21}(\lambda ) u = - v. \end{aligned}$$

Thus, eigenvalues of *L* outside the essential spectrum and their corresponding modes actually correspond to eigenfunctions of $S_1$, and contain a component in $\operatorname {Ker}(T)$. Therefore, to develop a true expansion for *L*, at least away from the essential spectrum, we should use the eigenfunctions of $S_1$.

To arrive at an expansion using the proper acoustic modes, we assume secular stability. Then $\gamma (a_2) = 0$ and $A_2^{1/2}$ is well defined on $D(A_2)$, with a nontrivial $\operatorname {Ker}(A_2^{1/2})$ coinciding with $\operatorname {Ker}(A_2)$. We let

34 $$\begin{aligned} B_2 = \begin{pmatrix} 0 &  \textrm{i}A_2^{1/2} \\ \textrm{i}A_2^{1/2} &  -2 \hat{R}_{\Omega } \end{pmatrix}, \end{aligned}$$

with

$$\begin{aligned} D(B_2) = D(a_2) \times D(a_2). \end{aligned}$$

It is immediate that $\textrm{i}B_2$ is *self-adjoint* on $H \times H$, equipped with the original inner product; indeed,

35 $$\begin{aligned} \left( B_2 \begin{pmatrix} u \\ v \end{pmatrix}, \begin{pmatrix} u' \\ v' \end{pmatrix}\right) = (\textrm{i}A_2^{1/2} v, u')_H + (\textrm{i}A_2^{1/2} u - 2 R_{\Omega } v, v')_H = -\left( \begin{pmatrix} u \\ v \end{pmatrix}, B_2 \begin{pmatrix} u' \\ v' \end{pmatrix}\right) . \end{aligned}$$

We introduce (noting the minus sign)

36 $$\begin{aligned} \widetilde{L}(\lambda ) = B_2 - \lambda \operatorname {Id}= \begin{pmatrix} -\lambda &  \textrm{i}{A}_2^{1/2} \\ \textrm{i}{A}_2^{1/2} &  -\lambda -2{R}_{\Omega } \end{pmatrix} \quad \text {and}\quad \widetilde{R}(\lambda ) = \widetilde{L}(\lambda )^{-1}. \end{aligned}$$

From Remark 4, it follows that $0 \notin \rho (L)$, so for $\lambda \in \rho (L)$ we can invert the previous equation to obtain

37 $$\begin{aligned} \widetilde{R}(\lambda ) = \widetilde{L}(\lambda )^{-1} = \begin{pmatrix} -\lambda ^{-1}(\operatorname {Id}- A_2^{1/2} R(\lambda ) A_2^{1/2}) &  -\textrm{i}{A}_2^{1/2} R(\lambda ) \\ -\textrm{i}R(\lambda ) {A}_2^{1/2} &  -\lambda R(\lambda ) \end{pmatrix}. \end{aligned}$$

On the other hand, if $\lambda \in \rho (\widetilde{L})$ we have an inverse

38 $$\begin{aligned} \widetilde{R}(\lambda ) = \begin{pmatrix} \widetilde{R}_{11}(\lambda ) &  \widetilde{R}_{12}(\lambda ) \\ \widetilde{R}_{12}(\lambda ) &  \widetilde{R}_{22}(\lambda ) \end{pmatrix}. \end{aligned}$$

Thus, the resolvents are related. If $\lambda \ne 0$, $R(\lambda ) = -\lambda ^{-1} R_{22}(\lambda )$. Remark 4 also implies that $0 \notin \rho (\widetilde{L})$ and so we see that $\rho (L) = \rho (\widetilde{L})$. Hence, the spectra are the same.

Suppose that $\lambda \in \sigma _{disc}(L) = \sigma _{disc}(\widetilde{L})$. Then a corresponding eigenfunction $(u, v) \in H \times H$ will satisfy

$$\begin{aligned} \textrm{i}A_2^{1/2} v = \lambda u,\quad \textrm{i}A_2^{1/2} u - 2 R_\Omega v = \lambda v. \end{aligned}$$

Restricting to acoustic modes, $v = 0$ is not possible since $\lambda \ne 0$. (That is, $\lambda = 0$ is an eigenvalue but does not correspond with an acoustic mode.) Thus, we can combine these formulae to obtain

39 $$\begin{aligned} L(\lambda ) v = 0,\quad u = \lambda ^{-1} \textrm{i}A_2^{1/2} v. \end{aligned}$$

Using the above calculations, we can introduce the Riesz projectors onto the space of acoustic modes, which are the spectrum of $S_1$. Indeed, let $\lambda \in \sigma (S_1)$ and $\Gamma _\lambda$ be a contour around surrounding $\lambda$ and no other part of $\sigma (B_2)$. Then consider the standard formula for the projection onto the eigenspace of $\lambda$

$$\begin{aligned} \widetilde{P}_\lambda = \frac{1}{2\pi i} \oint _{\Gamma _{\lambda }} \widetilde{R}(\mu ) \ \textrm{d} \mu . \end{aligned}$$

For more information on the definition of the Riesz projection and this contour integral, see [29]. we further let $\pi _v$ be projection onto the *v* component and define $P_\lambda = \pi _v \widetilde{P}_\lambda \pi ^*_v$. Then, using (37),

$$\begin{aligned} P_\lambda = -\frac{\lambda }{2\pi i} \oint _{\Gamma _\lambda } R(\mu ) \ \textrm{d} \mu . \end{aligned}$$

We can now use these projectors to define the projection onto the acoustic part of the spectrum, which is

$$\begin{aligned} E = \sum _{\lambda \in \sigma (S_1)} P_{\lambda }. \end{aligned}$$

We conclude that the projection onto the eigenspace of $\lambda$ for $\widetilde{L}$ gives a corresponding projection, by taking the *v* component as in (39), onto the space $\operatorname {Ker}(L(\lambda ))$ of an acoustic mode. This projection *E* shows it is possible to express the acoustic part of the wavefield as a sum of normal modes.

Using the above-mentioned Riesz projectors, we obtain a partial spectral decomposition of $\widetilde{R}_{22}(\lambda )$, namely into acoustic modes:

$$\begin{aligned} \widetilde{R}_{22}(\lambda )|_{acoustic} = \sum _{\omega \in \sigma (S_1)} \frac{P_\omega }{(\omega - \lambda )}. \end{aligned}$$

This induces a corresponding partial spectral decomposition of $R(\lambda )$ from (37):

$$\begin{aligned} R(\lambda )|_{acoustic} = \frac{1}{\lambda }\sum _{\omega \in \sigma (S_1)} \frac{P_\omega }{(\lambda - \omega )}, \end{aligned}$$

which is commonly used in computations.

## Inertia–gravity modes and essential spectrum

We now investigate the essential spectrum of *L*. Because $L_{11}^{-1}(\lambda )$ is compact and the $L_{ij}(\lambda )$ are bounded from Proposition 1, using Proposition 2 we have that

$$\begin{aligned} \sigma _{ess}(L) = \sigma _{ess}(L_{22}). \end{aligned}$$

Using the formula for $L_{22}$ given in Remark 2 and Lemma 4, this further reduces to

40 $$\begin{aligned} \sigma _{ess}(L) = \sigma _{ess}\left( \pi _2 \left( F(\lambda ) + N^2 \hat{g}_0' \hat{g}_0'^T \right) \pi _2^* \right) . \end{aligned}$$

Thus, referring to (14), we are led to consider the spectrum of

$$\begin{aligned} M(\lambda ) = \pi _2 (\lambda ^2 \textrm{Id}+2\lambda R_\Omega + N^2 \hat{g}_0' \hat{g}_0'^T) \pi _2^*: \operatorname {Ker(T)} \rightarrow \operatorname {Ker(T)}. \end{aligned}$$

Solutions $$u \in \operatorname {Ker}(T)$$ of

41 $$\begin{aligned} \partial _t^2 u + 2 \Omega \times \partial _t u + N^2 \hat{g}_0' \hat{g}_0'^T u = 0 \end{aligned}$$

are modes of *M*, referred to as inertia–gravity modes. Indeed, the restoring force of inertial modes is the Coriolis force, $2 \Omega \times \partial _t (\rho _0 u)$, while the restoring force of gravity modes is the buoyancy, $(\nabla \cdot \rho _0 u) g_0'$, which equals $N^2 \hat{g}_0' \hat{g}_0'^T \rho _0 u$ for $u \in \operatorname {Ker}(T)$. With both restoring forces, we speak of inertia–gravity modes.

We will precisely characterize the essential spectrum of *L* in Theorem 1, but must first introduce some notation and a definition. For convenience, let us define $P_\xi ^\perp = \sigma _p(\pi _2)$ defined by (22) which is the projection onto the space orthogonal to $\xi$. Also, for $\Omega \in \mathbb {R}^3$ let $\Omega _{\xi }$ be the component of $\Omega$ in the direction $\xi$ given by

$$\begin{aligned} \Omega _{\xi } = \frac{\xi \cdot \Omega }{|\xi |}. \end{aligned}$$

### Definition 2

For $x \in M$ and $\xi \in \mathbb {R}^3 {\setminus } \{0\}$, let $\sigma _{pt}(x,\xi )$ be the set of $\lambda \in \mathbb {C}$ such that

42 $$\begin{aligned} \mathbb {C}^3 \ni \eta \mapsto \lambda ^2 P_\xi ^\perp \eta + 2 \lambda P_\xi ^\perp ( \Omega \times P_\xi ^\perp \eta ) + N^2 (\hat{g}_0 \cdot P_\xi ^\perp \eta ) P_\xi ^\perp \hat{g}_0. \end{aligned}$$

has rank less than two (note that two is the largest possible rank due to $P_\xi ^\perp$).

In fact, the set $\sigma _{pt}(x,\xi )$ can be precisely characterized, which is done in the next lemma.

### Lemma 5

If $\lambda \in \sigma _{pt}(x,\xi )$, then $\lambda = 0$ or

43 $$\begin{aligned} \lambda = \pm i \sqrt{4 \Omega _\xi ^2 + N^2 |P_\xi ^\perp \hat{g}_0|^2}. \end{aligned}$$

### Proof

First, assume that $P_\xi ^\perp \hat{g}_0 \ne 0$ and set

44 $$\begin{aligned} \eta = a\ P_\xi ^\perp \hat{g}_0 + b\ \xi \times P_\xi ^\perp \hat{g}_0 \end{aligned}$$

where *a* and *b* are constants, not both equal to zero, to be determined. Calculation shows

$$\begin{aligned} \begin{aligned} P_\xi ^\perp \Big (\Omega \times P_\xi ^\perp (\xi \times P_\xi ^\perp \hat{g}_0 )\Big )&= P_\xi ^\perp \Big (\Omega \times (\xi \times P_\xi ^\perp \hat{g}_0 ) \Big )\\&= - |\xi | \Omega _\xi P_\xi ^\perp \hat{g}_0 \end{aligned} \end{aligned}$$

and

$$\begin{aligned} P_\xi ^\perp ( \Omega \times P_\xi ^\perp \hat{g}_0) = \frac{\Omega _\xi }{|\xi |} \xi \times P_\xi ^\perp \hat{g}_0. \end{aligned}$$

Therefore, if $\lambda \in \sigma _{pt}(x,\xi )$ then for some *a* and *b*

$$\begin{aligned} \Big ( \lambda ^2 a - 2 \lambda |\xi |\Omega _\xi b + N^2 |P_\xi ^\perp \hat{g}_0|^2 a \Big ) P_\xi \hat{g}_0 + \left( \lambda ^2 b + 2 \lambda \frac{\Omega _\xi }{|\xi |}a \right) \xi \times P_\xi ^\perp \hat{g}_0 = 0. \end{aligned}$$

Setting the two coefficients equal to zero, we see that either $\lambda = 0$ and $a = 0$ or

45 $$\begin{aligned} \lambda ^2 = -4 \Omega _\xi ^2 - N^2 |P_\xi ^\perp \hat{g}_0|^2 \end{aligned}$$

which completes the proof in this case. When $P_\xi ^\perp \hat{g}_0 = 0$, we choose arbitrary *w* orthogonal to $\xi$ and start with

$$\begin{aligned} \eta = a\ w + b\ \xi \times w \end{aligned}$$

instead of (44). A similar calculation gives $\lambda = 0$ or (45) in this case, and so the lemma is proved. $\square$

If $\lambda$ satisfies (43), then

46 $$\begin{aligned} \lambda ^2 = - \frac{1}{|\xi |^2} \Big (4 (\Omega \cdot \xi )^2 + N^2 |\xi |^2 - N^2 (\hat{g}_0' \cdot \xi )^2\Big ). \end{aligned}$$

The quantity in parentheses above is a quadratic form in $\xi$, and by determining the eigenvalues of the corresponding matrix we can determine the range of possible values of $\lambda ^2$. These eigenvalues are $N^2$ and

47 $$\begin{aligned} \beta _\pm = \frac{1}{2} \left( 4 |\Omega |^2 + N^2 \pm \sqrt{(N^2 + 4 |\Omega |^2)^2 - 16 (\Omega \cdot \hat{g}_0')^2 N^2} \right) . \end{aligned}$$

Therefore, the range of possible $\lambda ^2$ will be $-1$ times the interval between the minimum and maximum of these eigenvalues. If $N^2\ge 0$, then this range will be $\lambda ^2 \in -[\beta _-,\beta _+]$ which leads to $\lambda \in \pm i [\sqrt{\beta _-},\sqrt{\beta _+}]$. This agrees with the range of non-ellipticity of the Poincaré operator determined in [13]. In this case, it will be useful later for the proof Lemma 7 to note that $\sqrt{N^2} \in [\sqrt{\beta _-},\sqrt{\beta _+}]$. If $N^2 < 0$, then the range of possible values is $\lambda ^2 \in -[N^2,\beta _+]$, which gives $\lambda \in [-\sqrt{(-N^2)},\sqrt{(-N^2)}] \cup i [-\sqrt{\beta _+},\sqrt{\beta _+}]$. We combine these cases in the next lemma.

### Lemma 6

Let $\beta _\pm$ be given by (47). Then

48 $$\begin{aligned} \bigcup _{\xi \in \mathbb {R}^3 \setminus \{0\}} \sigma _{pt}(x,\xi ) = \bigcup _{\pm \in \{-1,1\}} \Bigg (\left[ -\sqrt{\max (0,-N^2)},\sqrt{\max (0,-N^2)}\right] \cup \pm i\left[ \sqrt{\max (0,\beta _-)},\sqrt{\beta _+} \right] \Bigg ). \end{aligned}$$

Furthermore, this set contains $\sqrt{-N^2}$.

**Fig. 1:**
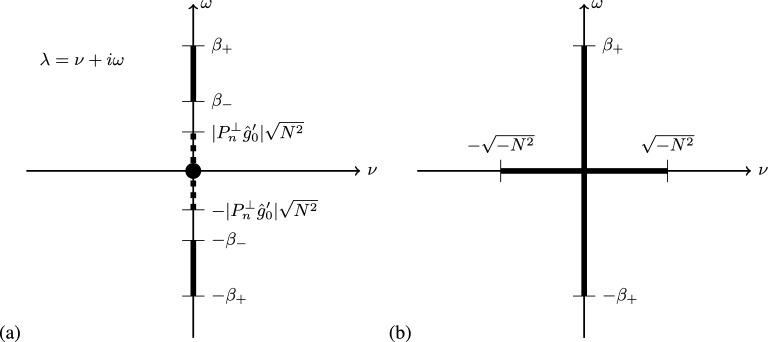
Solid black regions give the set (48) for fixed $x \in M$; in (**a**) on the left we see the case when $N^2\ge 0$ (note that this region includes the origin as indicated by the black dot), while in (**b**) on the right we see the case $N^2<0$. In reference to Theorem 1, the solid black regions are the areas where ellipticity fails at the point *x*, and appear for a single $x \in M$ in the union on the top line of (49). The dashed black region on the left is the set where the system (62) fails the Lopatinskii condition at the boundary but not in the interior, and appears for a single $x \in \partial M$ in the union on the second line of (49). Note that in (**a**) it is possible for the dashed set to intersect the solid set

We now present our characterization of the essential spectrum of *L*, which is the main result of this paper.

### Theorem 1

For $x\in \partial M$, let *n*(*x*) denote the inward pointing unit normal vector. The essential spectrum $\sigma _{ess}(L)$ is given by

49 $$\begin{aligned} \begin{aligned} \sigma _{ess}(L)&= \left( \bigcup _{x\in M,\ \pm \in \{-1,1\}} \left[ {}-\sqrt{\max (0,-N^2)},\sqrt{\max (0,-N^2)}\right] \cup \pm i\left[ \sqrt{\max (0,\beta _{-})},\sqrt{\beta _{+}} \right] \right) \\&\bigcup \left( \bigcup _{x\in \partial M} i|P_n^\perp \hat{g}_0'| \left[ {}-\sqrt{\max (0,N^2)},\sqrt{\max (0,N^2)}\right] \right) . \end{aligned} \end{aligned}$$

Before proving Theorem 1, we consider some special cases of the set in (48) from which we can obtain an upper bound on the essential spectrum in (49). If for some value of *x* we have $\Omega \cdot \hat{g}_0 = 0$, then from (47) we have

$$\begin{aligned} \beta _\pm = \min (0,4 |\Omega |^2+N^2), \ \max (0, 4|\Omega |^2 + N^2). \end{aligned}$$

Also, for general points $\beta _+ \le 4 |\Omega |^2 + N^2$. Therefore, considering (49), we see that the part of $\sigma _{ess}(L)$ along the imaginary axis must be contained in

$$\begin{aligned} i\left[ -\sqrt{4 |\Omega |^2 + \max (0,N_{\textrm{sup}}^2)},\sqrt{4 |\Omega |^2 + \max (0,N_{\textrm{sup}}^2)} \right] . \end{aligned}$$

On the other hand, directly from (49) we see that the part of $\sigma _{ess}(L)$ along the real axis must be contained in

$$\begin{aligned} \left[ {}-\sqrt{\max (0,-N_{\textrm{inf}}^2)},\sqrt{\max (0,-N_{\textrm{inf}}^2)}\right] \end{aligned}$$

Putting the previous remarks together, we see that

50 $$\begin{aligned} \sigma _{ess}(L) \subset \mathfrak {S}_{1} = \{ \nu + \textrm{i}\omega \in \mathbb {C}\,\ \nu = 0\ \text {and}\ \omega ^2 \le 4 |\Omega |^2 + \max (0,N^2_{\textrm{sup}})\ \text {or}\ \omega = 0\ \text {and}\ \nu ^2 \le \max (0,-N^2_{\textrm{inf}}) \}.\nonumber \\ \end{aligned}$$

**Fig. 2:**
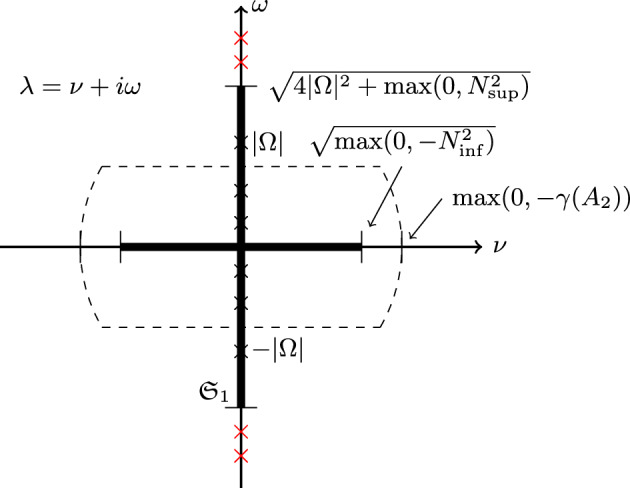
An illustration of the spectrum $\sigma (L)$ after Rogister and Valette [44]. The dark cross is the set $\mathfrak {S}_1$ which by Theorem 1, Lemma 5 and the discussion after the proof of that lemma, we have shown must contain the essential spectrum $\sigma _{ess}(L)$, but may in general be larger than the essential spectrum. By Proposition 3, the full spectrum $\sigma (L)$ is contained in the union of the imaginary axis and region surrounded by the dashed curve. The crosses on the imaginary axis are included to indicate eigenvalues, which could also occur within the dashed curve. The crosses which appear outside of the essential spectrum are red, indicating that they are part of $\sigma (S_1)$ which is the acoustic part of the spectrum

An illustration of the set $\mathfrak {S}_1$ is given in Figure 2.

In fact, the inclusion in (50) will always be an equality for the rotating self-gravitating truncated gas planets that we consider. This is because, using (5), calculation shows

$$\begin{aligned} \Omega \cdot g_0'(x) = -\Omega \cdot \int \limits _{M} G\frac{(x - x')}{|x-x'|^3} \rho ^0(x')\, \textrm{d} x'. \end{aligned}$$

Let $x_{max} \in \partial M$ maximize $\Omega \cdot x$. Then for any $x' \in \partial M$, $\Omega \cdot (x_{max}-x')\ge 0$ which implies $\Omega \cdot g_0'(x_{max}) \le 0$. Similarly, by choosing $x_{min}$ that minimizes $\Omega \cdot x$ we can show $\Omega \cdot g_0'(x_{min}) \ge 0$. Therefore, by continuity, $\Omega \cdot \hat{g}'_0 = 0$ somewhere in *M* and so at this point $\beta _- = 0$. Because of this, we can conclude equality in (50). On the other hand, in the f-plane approximation as considered in [13], $\Omega \cdot \hat{g}_0'$ is constant and so never vanishes and (50) is a proper inclusion.

### Remark 5

For a neutrally buoyant planet, $\tilde{s} = 0$ and $N^2 = 0$. Then the relevant operator $M(\lambda )$ reduces to the Poincaré operator [42]. In the polytropic model, the planet is neutrally buoyant. In the case that $N^2 = 0$, Theorem 1 also gives the essential spectrum of $2 \pi _2 R_\Omega \pi _2^*$ which, by Lemma 5, is $i[-|\Omega |,|\Omega |]$. As observed in [48, Page 138], this interval contains the spectrum of $2 \pi _2 R_\Omega \pi _2^*$, and so must in fact be equal to the full spectrum.

### Proof of Theorem 1

We begin by proving the inclusion,

51 $$\begin{aligned} \begin{aligned} \sigma _{ess}(L)&\supset \bigcup _{x\in M,\ \pm \in \{-1,1\}} \left[ -\sqrt{\max (0,-N^2)},\sqrt{\max (0,-N^2)}\right] \cup \pm i\left[ \sqrt{\max (0,\beta _-)},\sqrt{\beta _+} \right] \\&= \bigcup _{(x,\xi )\in M\times \mathbb {R}^3 \setminus \{0\}} \sigma _{pt}(x,\xi ). \end{aligned} \end{aligned}$$

Our method for this step is inspired by [10, Theorem 2.1], which considers a similar but simpler problem for a scalar function. Suppose that $\lambda \in \mathbb {C}$ is contained in $\sigma _{pt}(x_0,\xi _0)$ such that $x_0 \in M^{int}$. Thus, there exists nonzero $\eta$ orthogonal to $\xi _0$ such that

52 $$\begin{aligned} \lambda ^2 P_{\xi _0} \eta + 2 \lambda P_{\xi } (\Omega \times P_{\xi _0} \eta ) + N^2 (P_{\xi _0} \hat{g}_0 \cdot P_{\xi _0} \eta ) P_{\xi _0} \hat{g}_0 = 0. \end{aligned}$$

Then, for any $\epsilon >0$, choose a neighborhood $U \subset M^{int}$ of $x_0$ such that at all $x \in U$

$$\begin{aligned} |\lambda ^2 P_{\xi _0} \eta + 2 \lambda P_{\xi _0} (\Omega \times P_{\xi _0} \eta ) + N^2 (P_{\xi _0} \hat{g}_0 \cdot P_{\xi _0} \eta ) P_{\xi _0} \hat{g}_0| < \epsilon . \end{aligned}$$

Let $\phi \in C_c^\infty (U)$ be such that $\Vert \phi \Vert _{L^2(\rho ^0 \textrm{d}x)} = 1$ and consider

$$\begin{aligned} u(x) = \eta \phi (x) e^{it x \cdot \xi _0}. \end{aligned}$$

Considering the Fourier transform, we can see that as $t \rightarrow \infty$, *u* converges to zero weakly. Since $\pi _2$ is a pseudodifferential operator with principal symbol given by (22), using the fact that $\xi _0$ is orthogonal to $\eta$, we have

$$\begin{aligned} \pi _2(u)(x) = \phi (x) e^{it x \cdot \xi _0} \eta + O\left( \frac{1}{t} \right) . \end{aligned}$$

Therefore, for *t* sufficiently large $\Vert \pi _2(u)\Vert _{L^2(\rho _0 \textrm{d}x)^3}>C>0$ where *C* is a constant independent of *t*. Also, since $\pi _2$ is continuous $\pi _2(u)$ converges weakly to zero as $t \rightarrow \infty$. Let us set

$$\begin{aligned} v = \frac{\pi _2(u)}{\Vert \pi _2(u) \Vert _{H}} \in \operatorname {Ker}(T). \end{aligned}$$

Then

$$\begin{aligned} M(\lambda )v = \frac{1}{\Vert \pi _2(u) \Vert _{H}}\pi _2 (\lambda ^2 \textrm{Id}+2\lambda R_\Omega + N^2 \hat{g}_0' \hat{g}_0'^T) \pi _2 u \end{aligned}$$

and the operator on the right side is a pseudodifferential operator with principal symbol given by the map (42). Thus,

$$\begin{aligned} M(\lambda )v = \frac{1}{\Vert \pi _2(u) \Vert _{H}}\Big (\lambda ^2 P_{\xi _0} \eta + 2 \lambda P_{\xi _0} (\Omega \times P_{\xi _0} \eta ) + N^2 (P_{\xi _0} \hat{g}_0 \cdot P_{\xi _0} \eta ) P_{\xi _0} \hat{g}_0 \Big ) \phi (x) e^{it x \cdot \xi _0} + O\left( \frac{1}{t} \right) \end{aligned}$$

and so by taking *t* sufficiently large

$$\begin{aligned} \Vert M(\lambda ) v \Vert _{L^2(\rho _0 \textrm{d}x)^3} \le \frac{2}{\Vert \pi _2(u) \Vert _{H}} \epsilon . \end{aligned}$$

Since $\epsilon >0$ was arbitrary we see that $M(\lambda ) v$ converges to zero strongly and so *v* defines a Weyl sequence. Therefore, $\lambda \in \sigma _{ess}(M) = \sigma _{ess}(L)$. This proves $\sigma _{pt}(x_0,\xi _0) \subset \sigma _{ess}(L)$ for $x_0 \in M^{int}$. Since the essential spectrum is closed and (43) is a continuous function of *x* once ± is chosen, for $x_0 \in \partial M$ we can take a limit from $M^{int}$ to show $\sigma _{pt}(x_0,\xi ) \subset \sigma _{ess}(L)$. This completes the proof of (51).

To complete the proof, our method will be to introduce a certain system of PDEs, then show that this system satisfies the Lopatinskii conditions [1], see also [46, Chapter 5, Proposition 11.9], if and only if $\lambda$ is in the complement of the right side of (49). When the Lopatinskii conditions are satisfied, the system is a Fredholm operator which implies $M(\lambda )$ is also Fredholm. Therefore, in this case $\lambda \in \sigma _{ess}(L)^c$ which will establish the right inclusion of (49). The Lopatinskii conditions fail if either the system is not elliptic in the interior, or at the boundary. As we will see, interior ellipticity of the system is equivalent to

53 $$\begin{aligned} \lambda \in \left( \bigcup _{(x,\xi )\in M\times \mathbb {R}^3 \setminus \{0\}} \sigma _{pt}(x,\xi ) \right) ^c. \end{aligned}$$

We have already shown that failure of this condition leads to existence of a Weyl sequence. Assuming interior ellipticity, we will show that boundary ellipticity is equivalent to

$$\begin{aligned} \lambda \in \left( \bigcup _{x\in \partial M} i |P_n\left[ -\sqrt{\max (0,N^2)},\sqrt{\max (0,N^2)}\right] \right) ^c \end{aligned}$$

We will show that failure of this condition also leads to existence of a Weyl sequence, which will complete the proof. Let us begin now deriving the PDE system.

For any $v \in H$ let us consider the decomposition given by Lemma 2, which can be written as

$$\begin{aligned} v = w + T^* \varphi \end{aligned}$$

where $w \in \operatorname {Ker}(T)$ and $\varphi \in H^1(M)$. Let us further decompose *w* according the standard Helmholtz decomposition as

$$\begin{aligned} w = \nabla \times (\rho _0 w_v) + \nabla \varphi _v \end{aligned}$$

where $\varphi _v \in H^1(M)$ and the vector potential $\rho _0 w_v$ is in the space

$$\begin{aligned} H_{\textrm{Curl},0}(M) = \{ u \in L^2(\rho _0 \textrm{d}x) \, \ \nabla \times u \in L^2(\rho _0 \textrm{d}x), \ n\times u|_{\partial M} = 0 \}, \end{aligned}$$

while also satisfying

$$\begin{aligned} \nabla \cdot (\rho _0 w_v) = 0. \end{aligned}$$

Given that *M* is a ball, a unique such decomposition exists (see [2, Section 3]). Let us set $\rho _0 z_v = \nabla \varphi _v$ which must then satisfy

$$\begin{aligned} \nabla \times (\rho _0 z_v) = 0. \end{aligned}$$

Then $w \in \operatorname {Ker}(T)$ is equivalent to

$$\begin{aligned} \nabla \cdot (\rho _0 z_v) + \frac{g_0'}{c^2} \cdot \nabla \times (\rho _0 w_v) + \frac{\rho _0 g_0'}{c^2} \cdot z_v = 0, \quad n \cdot z_v|_{\partial M} = 0. \end{aligned}$$

Now, suppose that $u \in \operatorname {Ker}(T)$ satisfies

54 $$\begin{aligned} M(\lambda ) u = f. \end{aligned}$$

As described above for *v*, there will be $w_u$ and $z_u$ such that

$$\begin{aligned} u = \nabla \times (\rho _0 w_u) + \rho _0 z_u \end{aligned}$$

where

55 $$\begin{aligned} \nabla \times (\rho _0 z_u)&= 0, \end{aligned}$$

56 $$\begin{aligned} \nabla \cdot (\rho _0 w_u)&= 0, \end{aligned}$$

57 $$\begin{aligned} \nabla \cdot (\rho _0 z_u) + \frac{g_0'}{c^2} \cdot \nabla \times (\rho _0 w_u) + \frac{\rho _0 g_0'}{c^2} \cdot z_u&= 0 , \end{aligned}$$

58 $$\begin{aligned} n \cdot z_u|_{\partial M}&= 0, \end{aligned}$$

59 $$\begin{aligned} n \times w_u|_{\partial M}&= 0. \end{aligned}$$

We comment that the same equations (55)-(59) will hold for $w_v$ and $z_v$ constructed above for arbitrary *v*. Indeed, let $V(\lambda ) = \lambda ^2 \textrm{I} + 2 \lambda R_\Omega + N^2 \hat{g}_0'\hat{g}_0'^T$ and

$$\begin{aligned} v = V(\lambda ) u \end{aligned}$$

so that $f = \pi _2 v$. These equations become

60 $$\begin{aligned} \nabla \times (\rho _0 w_v) + \rho _0 z_v + T^* \varphi _v&= V(\lambda ) ( \nabla \times (\rho _0 w_u) + \rho _0 z_u), \end{aligned}$$

61 $$\begin{aligned} f&= \nabla \times (\rho _0 w_v) + \rho _0 z_v. \end{aligned}$$

To make the system of equations elliptic, we will also add several potentials $\psi _u$, $\psi _v$ and $\widetilde{\varphi }$. Setting these equal to zero, we find that the following system is satisfied.

62 $$\begin{aligned} \begin{pmatrix} \frac{g_0'^T}{c^2} \nabla \times \rho _0 &  \nabla \cdot \rho _0 + \frac{\rho _0 g_0'^T}{c^2} &  0 &  0 &  0 &  0 &  0 &  0\\ 0 &  \nabla \times \rho _0 &  \nabla \rho _0 &  0 &  0 &  0 &  0 &  0\\ \nabla \cdot \rho _0 &  0 &  0 &  0 &  0 &  0 &  0 &  0\\ 0 &  0 &  0 &  \frac{g_0'^T}{c^2}\nabla \times \rho _0 &  \nabla \cdot \rho _0 + \frac{\rho _0 g_0'^T}{c^2} &  0 &  0 &  0\\ 0 &  0 &  0 &  0 &  \nabla \times \rho _0 &  \nabla \rho _0 &  0 &  0\\ 0 &  0 &  0 &  \nabla \cdot \rho _0 &  0 &  0 &  0 &  0\\ V(\lambda ) \nabla \times \rho _0 &  V(\lambda )\rho _0 &  0 &  - \nabla \times \rho _0 &  -\rho _0 I &  0 &  -T^* &  0\\ 0 &  0 &  0 &  \nabla \times \rho _0 &  \rho _0 I &  0 &  0 &  -T^* \end{pmatrix} \begin{pmatrix} w_u\\ z_u\\ \psi _u\\ w_v\\ z_v\\ \psi _v\\ \varphi _v\\ \widetilde{\varphi } \end{pmatrix} = \begin{pmatrix} 0\\ 0\\ 0\\ 0\\ 0\\ 0\\ 0\\ f\\ \end{pmatrix}, \end{aligned}$$

$$\begin{aligned} n\times w_u|_{\partial M} = n\times w_v|_{\partial M} = 0,\ n\cdot z_u|_{\partial M} = n \cdot z_v|_{\partial M} = \psi _u|_{\partial M} = \psi _v|_{\partial M} = 0. \end{aligned}$$

In Lemma 7, we show that the system (62) satisfies the Lopatinskii conditions when $\lambda$ is in the complement of the right side of the (49). Therefore, for such $\lambda$ and by [46, Chapter 5, Proposition 11.16], when acting on $H^1(M)^{16}$ the corresponding operator is Fredholm. Considering that whenever (54) is satisfied we have (62), we therefore conclude that $M(\lambda )$ is also Fredholm in this case. Thus $\lambda \in \sigma _{ess}(M)^c = \sigma _{ess}(L)^c$ which shows the right inclusion for (49).

All that remains is to show that when

63 $$\begin{aligned} \lambda \in \left( \bigcup _{x\in \partial M} i|P_n\hat{g}_0'|\left[ -\sqrt{\max (0,N^2)},\sqrt{\max (0,N^2)}\right] \right) \bigcap \left( \bigcup _{(x,\xi )\in M\times \mathbb {R}^3 \setminus \{0\}} \sigma _{pt}(x,\xi ) \right) ^c, \end{aligned}$$

$\lambda \in \sigma _{ess}(M)$. For this last step, it is necessary to use the details of the computation checking the Lopatinskii condition, and so it is also proved in Lemma 7. Therefore, using Lemma 7 the proof is complete. $\square$

The next lemma is the key technical step in the proof of Theorem 1.

### Lemma 7

Suppose that $\lambda$ is in the complement of the right side of (49). Then the system (62) satisfies the Lopatinskii conditions. Furthermore, suppose (63). Then $\lambda \in \sigma _{ess}(M)$.

### Proof

Let the operator on the left side of (62) be labeled $\mathcal {M}( \lambda )$. Also suppose we collect the relevant operators for the boundary conditions in one large matrix

64 $$\begin{aligned} \mathcal {B} = \begin{pmatrix} n\times &  0 &  0 &  0 &  0 &  0 &  0 &  0\\ 0 &  n^T &  0 &  0 &  0 &  0 &  0 &  0\\ 0 &  0 &  1 &  0 &  0 &  0 &  0 &  0\\ 0 &  0 &  0 &  n\times &  0 &  0 &  0 &  0\\ 0 &  0 &  0 &  0 &  n^T &  0 &  0 &  0\\ 0 &  0 &  0 &  0 &  0 &  1 &  0 &  0 \end{pmatrix}. \end{aligned}$$

The principal symbol of $\mathcal {M}(\lambda )$ is

65 $$\begin{aligned} \sigma _p(\mathcal {M}(\lambda )) = i \rho _0 \begin{pmatrix} \frac{g_0^T}{c^2} \xi \times &  \xi ^T &  0 &  0 &  0 &  0 &  0 &  0 \\ 0 &  \xi \times &  \xi &  0 &  0 &  0 &  0 &  0\\ \xi ^T &  0 &  0 &  0 &  0 &  0 &  0 &  0\\ 0 &  0 &  0 &  \frac{g_0^T}{c^2} \xi \times &  \xi ^T &  0 &  0 &  0\\ 0 &  0 &  0 &  0 &  \xi \times &  \xi &  0 &  0 \\ 0 &  0 &  0 &  \xi ^T &  0 &  0 &  0 &  0\\ V(\lambda ) \xi \times &  0 &  0 &  -\xi \times &  0 &  0 &  \xi &  0\\ 0 &  0 &  0 &  \xi \times &  0 &  0 &  0 &  \xi \end{pmatrix}. \end{aligned}$$

This can be shown to be invertible if $V(\lambda )$ is invertible when projected onto the space orthogonal to $\xi$. Indeed, let us define

$$\begin{aligned} V_{\xi _\perp \xi _\perp }(\lambda ) = P_\xi ^\perp V(\lambda ) P_\xi ^\perp , \ V_{\xi \xi _\perp }(\lambda ) = P_\xi V(\lambda ) P_\xi ^\perp \end{aligned}$$

where $P_{\xi }$ is the projection onto the span of $\xi$ and $P_\xi ^\perp$ the projection onto the space orthogonal to $\xi$. The condition (53) is equivalent to invertibility of $\widetilde{V}_\xi (\lambda ) = V_{\xi _\perp \xi _\perp }(\lambda ) + P_\xi$ at all points $(x,\xi ) \in M \times (\mathbb {R}^3 {\setminus } \{0\})$. In the sequel we will suppress the dependence on $\lambda$ to ease the notation. When it exists, the inverse of $\sigma _p(\mathcal {M})$ is given by

66 $$\begin{aligned} \sigma _p(\mathcal {M})^{-1} = -\frac{i}{\rho _0 |\xi |^2} \begin{pmatrix} 0 &  0 &  \xi &  0 &  0 &  0 &  - \xi \times \widetilde{V}^{-1}_\xi &  - \xi \times \widetilde{V}^{-1}_\xi \\ \xi &  - \xi \times &  0 &  0 &  0 &  0 &  -\xi \frac{g_0'^T}{c^2} P_\xi ^\perp \widetilde{V}^{-1}_{\xi } &  -\xi \frac{g_0'^T}{c^2} P_\xi ^\perp \widetilde{V}^{-1}_{\xi }\\ 0 &  \xi ^T &  0 &  0 &  0 &  0 &  0 &  0\\ 0 &  0 &  0 &  0 &  0 &  \xi &  0 &  -\xi \times \\ 0 &  0 &  0 &  \xi &  -\xi \times &  0 &  0 &  -\xi \frac{g_0'^T}{c^2}P_\xi ^\perp \\ 0 &  0 &  0 &  0 &  \xi ^T &  0 &  0 &  0\\ 0 &  0 &  0 &  0 &  0 &  0 &  \xi ^T (I - V_{\xi \xi _\perp }) \widetilde{V}^{-1}_\xi &  -\xi ^T V_{\xi \xi _\perp } \widetilde{V}^{-1}_\xi P_\xi ^\perp \\ 0 &  0 &  0 &  0 &  0 &  0 &  0 &  \xi ^T \end{pmatrix}. \end{aligned}$$

Let us consider the Lopatinskii condition in boundary normal coordinates $(\widetilde{x},x^3)$ where we freeze all coefficients at the central point where the Euclidean metric is the identity and write *n* for the inward pointing unit normal vector. Without loss of generality we assume the central point is the origin. The condition is that there is a unique nonzero bounded solution of the system

67 $$\begin{aligned} \sigma _p(\mathcal {M})(\widetilde{\xi }+ n D_3) U = 0, \quad \mathcal {B}U = \eta \end{aligned}$$

for any nonzero real $\widetilde{\xi } \in \mathbb {R}^{3}$ orthogonal to *n* and $\eta \in \mathbb {C}^{8}$. Assuming $\lambda \in \sigma _{pt}((\widetilde{x},x^3),n)^c$, the ODE (67) is equivalent to

$$\begin{aligned} \frac{\textrm{d}U}{\textrm{d}x^3} = -\sigma _p(\mathcal {M})\left( \frac{n}{i}\right) ^{-1}\sigma _p(\mathcal {M})(\widetilde{\xi })U \end{aligned}$$

and checking the condition amounts to analyzing the eigenvalues and eigenvectors of the matrix on the right side of this equation. Let us label this matrix

$$\begin{aligned} K = -\sigma _p(\mathcal {M})\left( \frac{n}{i}\right) ^{-1}\sigma _p(\mathcal {M})(\widetilde{\xi }). \end{aligned}$$

Note that, because of (66), when the ellipticity condition is satisfied at the boundary *K* cannot have any eigenvalues with zero real part. Considerable calculation shows that the eigenvalues of *K* are $\pm |\widetilde{\xi }|$ each with algebraic multiplicity 7 and

$$\begin{aligned} \begin{aligned} \alpha _\pm&= i|\widetilde{\xi }|\Bigg ( n^TV_{nn_\perp } \widetilde{V}_n^{-1} \hat{\xi }+\hat{\xi }^T\widetilde{V}_n^{-1}V_{n_\perp n} n \\&\hspace{72.26999pt}\mp \sqrt{(n^TV_{nn_\perp } \widetilde{V}_n^{-1} \hat{\xi }-\hat{\xi }^T\widetilde{V}_n^{-1}V_{n_\perp n} n)^2 - 4(\hat{\xi }\widetilde{V}_n^{-1}\hat{\xi })n^T(V_{nn}-V_{nn_\perp }\widetilde{V}_n^{-1}V_{n_\perp n})n} \Bigg ) /2 \end{aligned} \end{aligned}$$

with multiplicity 1, or possibly $\pm |\widetilde{\xi }|$ with multiplicity 8 if $\alpha _\pm = \pm |\widetilde{\xi }|$. Note that, provided (53) holds, $\alpha _\pm$ must have nonzero real part by the ellipticity condition.

Let us introduce the notation

$$\begin{aligned} \hat{\xi } = \frac{\widetilde{\xi }}{|\widetilde{\xi }|}, \quad n_\perp = \hat{\xi } \times n. \end{aligned}$$

Eigenvectors for $\pm |\widetilde{\xi }|$ are

$$\begin{aligned} U_{1,\pm } = \begin{pmatrix} n \pm i \hat{\xi }\\ 0\\ 0\\ 0\\ 0\\ 0\\ 0\\ 0 \end{pmatrix},\ U_{2,\pm } = \begin{pmatrix} 0\\ n \pm i \hat{\xi }\\ 0\\ 0\\ 0\\ 0\\ 0\\ 0 \end{pmatrix}, U_{3,\pm } = \begin{pmatrix} 0\\ n_\perp \\ \pm i \\ 0\\ 0\\ 0\\ 0\\ 0 \end{pmatrix}, \\ U_{4,\pm } = \begin{pmatrix} 0\\ 0\\ 0\\ n\pm i\hat{\xi }\\ 0\\ 0\\ 0\\ 0, \end{pmatrix},\ U_{5,\pm } = \begin{pmatrix} 0\\ 0\\ 0\\ 0\\ n\pm i \hat{\xi }\\ 0\\ 0\\ 0, \end{pmatrix},\ U_{6,\pm } = \begin{pmatrix} 0\\ 0\\ 0\\ 0\\ n_\perp \\ \pm i\\ 0\\ 0, \end{pmatrix} \end{aligned}$$

and there are either eigenvectors or generalized eigenvectors for $\pm |\widetilde{\xi }|$ of the form

$$\begin{aligned} U_{7,\pm } = \begin{pmatrix} 0\\ 0\\ 0\\ n_\perp \\ a_{7,\pm }n+ b_{7,\pm }\hat{\xi }\\ 0\\ \pm i\\ \mp i \end{pmatrix} \end{aligned}$$

for some constants $a_{7,\pm }$, $b_{7,\pm } \in \mathbb {C}$. Finally, either eigenvectors for $\lambda _\pm$ or generalized eigenvectors for $\pm |\tilde{\xi }|$ are given by

$$\begin{aligned} U_{8,\pm } = \begin{pmatrix} 2(\hat{\xi }^T\widetilde{V}_n^{-1} \hat{\xi }) n_\perp + a_{8, \pm } n + b_{8,\pm }\hat{\xi }\\ c_{8, \pm } n + d_{8,\pm }\hat{\xi }\\ 0\\ 0\\ 0\\ 0\\ (n^TV_{nn_\perp } \widetilde{V}_n^{-1} \hat{\xi }-\hat{\xi }^T\widetilde{V}_n^{-1}V_{n_\perp n} n) \hspace{274.6262pt}\\ \hspace{72.26999pt}\pm \sqrt{(n^TV_{nn_\perp } \widetilde{V}_n^{-1} \hat{\xi }-\hat{\xi }^T\widetilde{V}_n^{-1}V_{n_\perp n} n)^2 - 4 (\hat{\xi }^T\widetilde{V}_n^{-1}\hat{\xi })n^T(V_{nn}-V_{nn_\perp }\widetilde{V}_n^{-1}V_{n_\perp n})n }\\ 0 \end{pmatrix} \end{aligned}$$

for some constants $a_{8,\pm }$, $b_{8,\pm }$, $c_{8,\pm }$, $d_{8,\pm } \in \mathbb {C}$. For the Lopatinskii condition we must restrict to the generalized eigenspace corresponding to eigenvalues with negative real part. Thus, existence of a unique bounded solution of (67) is equivalent to a unique solution $(a_1, \ldots \, a_8) \in \mathbb {C}^8$ of the system

$$\begin{aligned} \mathcal {B}\sum _{j=1}^8 a_j U_{j,-} = \eta . \end{aligned}$$

Using (64) and the equations for $U_{j,-}$ above we see that this linear system will have a unique solution if and only if $\hat{\xi }^T \widetilde{V}^{-1}_n \hat{\xi } \ne 0$. Calculation shows

$$\begin{aligned} \widetilde{V}_{n}^{-1} = P_n + \frac{1}{\lambda ^4 + \lambda ^2 (N^2 |P_n^\perp \hat{g}_0'|^2 + 4 \Omega _n^2)} \Big (\lambda ^2 P_n^\perp -2\lambda \Omega _n R_n + N^2 |P_{n}^{\perp }\hat{g}_0'|^2 P_{n}^\perp P_{(P_{n}^{\perp }\hat{g}_0')}^\perp P_{n}^\perp \Big ), \end{aligned}$$

and so, since $\hat{\xi }$ is orthogonal to *n*,

$$\begin{aligned} \hat{\xi }^T \widetilde{V}^{-1}_n \hat{\xi } = \frac{\lambda ^2 + N^2 |P_{n}^{\perp }\hat{g}_0'|^2 \hat{\xi }^T P_{(P_{n}^{\perp }\hat{g}_0')}^\perp \hat{\xi }}{\lambda ^4 + \lambda ^2 (N^2 |P_n^\perp \hat{g}_0'|^2 + 4 \Omega _n^2)} \end{aligned}$$

Therefore, for $\lambda$ satisfying the interior ellipticity condition (53), the Lopatinskii condition fails if and only if

$$\begin{aligned} \lambda ^2 = -N^2 |P_{n}^{\perp }\hat{g}_0'|^2 \hat{\xi }^T P_{(P_{n}^{\perp }\hat{g}_0')}^\perp \hat{\xi }. \end{aligned}$$

If $|P_n^\perp g_0'| \ne 0$, then $\hat{\xi }^T P_{(P_{n}^{\perp }\hat{g}_0')}^\perp \hat{\xi }$ takes all values in [0, 1] while if $|P_n^\perp g_0'|=0$ then the right side of this equation is always equal zero. Therefore, we see that the range of possible values of $\lambda$ satisfying this equation is $|P_n^\perp \hat{g}_0'| [-\sqrt{-N^2},\sqrt{-N^2}]$. If $N^2<0$, this is already contained in the interior part of the essential spectrum given by the first line of (49). If $N^2\ge 0$, this interval will not be contained in the interior part of the essential spectrum and is given, for a single $x \in \partial M$, by the second line in (49) (see Fig. 1a).

It remains to show that given (63), $\lambda \in \sigma _{ess}(M)$. We will do this by showing the existence of a Weyl sequence. Indeed, by the calculations above, we see that when the Lopatinskii condition fails, for some $\widetilde{\xi }$ orthogonal to *n* if we set $\zeta = U_{8,-} - i b_{8,-} U_{1,-} - i d_{8,-} U_{2,-}$, then we have

$$\begin{aligned} \mathcal {B}\zeta = 0. \end{aligned}$$

Since $\zeta$ is composed of eigenvectors for eigenvalues with negative real part, there will be a corresponding nonzero bounded solution $U_\zeta$ of the ODE in (67) with $U_{\zeta }( \widetilde{\xi },x^3 = 0) = \zeta$. Given $\epsilon >0$, let us choose a neighborhood $\Omega$ of *x* sufficiently small so that all coefficients of operator $\mathcal {M}$ vary by at most $\epsilon$ within the neighborhood, and let $\phi \in C_c^\infty (\Omega )$. Then we set

68 $$\begin{aligned} \mathcal {U}(x) = \phi (x) e^{i t \widetilde{x} \cdot \widetilde{\xi }} U_\zeta (\widetilde{\xi },tx^3) \end{aligned}$$

which is in $H^1(M)^{16}$. With this choice of $\mathcal {U}$ we have

$$\begin{aligned} \begin{aligned} \mathcal {M}(\lambda ) \mathcal {U}(x)&= \mathcal {M}(\lambda )|_{x = 0} \mathcal {U} + \epsilon \mathcal {O}(t)\\&= it \phi (x) \sigma _p(\mathcal {M})|_{x =0}(\widetilde{\xi } + n D_3)\mathcal {U} + \epsilon \mathcal {O}(t) + \mathcal {O}(1)\\&= \epsilon \mathcal {O}(t) + \mathcal {O}(1) \end{aligned} \end{aligned}$$

as $t \rightarrow \infty$ with norm $H^1(M)^{16}$. Now let $w_u$ and $z_u$ be the corresponding components of $\mathcal {U}$. Since $\hat{\xi }^T \tilde{V}_n^{-1} \hat{\xi } = 0$, in the case when $\alpha _- \ne -|\widetilde{\xi }|$ these are explicitly given by

69 $$\begin{aligned} \begin{aligned} w_u&= e^{t(x^3\alpha _-+ i\widetilde{x}\cdot \widetilde{\xi })}(a_{8,-} n + b_{8_,-}\hat{\xi }) -ib_{8,-} e^{t(-x^3|\widetilde{\xi }| + i\widetilde{x}\cdot \widetilde{\xi })}(n-i\hat{\xi }),\\ z_u&= e^{t(x^3\alpha _-+ i\widetilde{x}\cdot \widetilde{\xi })}(c_{8,-} n + d_{8_,-}\hat{\xi }) -id_{8,-} e^{t(-x^3|\widetilde{\xi }| + i\widetilde{x}\cdot \widetilde{\xi })}(n-i\hat{\xi }). \end{aligned} \end{aligned}$$

In the case that $\alpha _- = -|\widetilde{\xi }|$ and $U_{8,-}$ is a generalized eigenvector, these are replaced by

70 $$\begin{aligned} \begin{aligned} w_u&= e^{t(-x^3|\widetilde{\xi }|+ i\widetilde{x}\cdot \widetilde{\xi })}(a_{8,-}-ib_{8,-}) n +tx^3 e^{t(-x^3|\widetilde{\xi }| + i\widetilde{x}\cdot \widetilde{\xi })}(n-i\hat{\xi }),\\ z_u&= e^{t(-x^3|\widetilde{\xi }|+ i\widetilde{x}\cdot \widetilde{\xi })}(c_{8,-}-id_{8,-}) n +tx^3 e^{t(-x^3|\widetilde{\xi }| + i\widetilde{x}\cdot \widetilde{\xi })}(n-i\hat{\xi }). \end{aligned} \end{aligned}$$

Then, considering the first component of (62), we have $\nabla \times (\rho _0 w_u) + \rho _0 z_u \in D(T)$ and

$$\begin{aligned} T (\nabla \times (\rho _0 w_u) + \rho _0 z_u) = \epsilon \mathcal {O}(t) + \mathcal {O}(1). \end{aligned}$$

By the construction of $\pi _2$ described in Lemma 3, we have

$$\begin{aligned} \pi _2(\nabla \times (\rho _0 w_u) + \rho _0 z_u) = \nabla \times (\rho _0 w_u) + \rho _0 z_u + \epsilon \mathcal {O}(t) + \mathcal {O}(1) \end{aligned}$$

with the norm *H*. With this in mind, let us set $u = \pi _2(\nabla \times (\rho _0 w_u) + \rho _0 z_u) \in \operatorname {Ker}(T)$, and consider $M(\lambda )u$. Using the last and second to last lines in (62) and the fact that most components of $\mathcal {U}$ are zero, we obtain

$$\begin{aligned} M(\lambda )u = \epsilon \mathcal {O}(t) + \mathcal {O}(1). \end{aligned}$$

To construct a Weyl sequence, we need to normalize *u*, and so we consider $\Vert \nabla \times (\rho _0 w_u) + \rho _0 z_u\Vert _H$. In the case $U_{8,-}$ is not a generalized eigenvector, using (69) we see that

$$\begin{aligned} \nabla \times (\rho _0 w_u) + \rho _0 z_u = t e^{t(x^3\alpha _-+ i\widetilde{x}\cdot \widetilde{\xi })}\left( -\frac{\alpha _-}{|\widetilde{\xi }|} b_{8,-} + i a_{8,-} \right) |\widetilde{\xi }| n_\perp + t e^{t(-x^3|\widetilde{\xi }|+ i\widetilde{x}\cdot \widetilde{\xi })} i b_{8,-}|\widetilde{\xi }| n_\perp + \mathcal {O}(1). \end{aligned}$$

Since, from the calculation constructing $U_{8,-}$, we know that $a_{8,-}$ and $b_{8,-}$ cannot simultaneously vanish, from this last formula we see that

$$\begin{aligned} \Vert u\Vert _H = \Vert \nabla \times (\rho _0 w_u) + \rho _0 z_u\Vert _H + \epsilon \mathcal {O}(t) + \mathcal {O}(t) \approx \mathcal {O}(t). \end{aligned}$$

By this notation, we mean that $\Vert u\Vert _H$ is bounded below by *Ct* as $t\rightarrow \infty$ for some constant $C>0$. A similar calculation beginning with (70), omitted here, proves the same result when $U_{8,-}$ is a generalized eigenvector. Therefore

$$\begin{aligned} M(\lambda ) \frac{u}{\Vert u\Vert _H} = \epsilon \mathcal {O}(1) + \mathcal {O}(t^{-1}) \end{aligned}$$

and so by choosing *t* sufficiently large we can obtain a sequence $v_\epsilon = u/\Vert u\Vert _H \in \operatorname {Ker}(T)$ with *H*-norm equal to one and such that $M(\lambda )v_\epsilon \rightarrow 0$ as $\epsilon \rightarrow 0$. Because of the oscillatory nature of (68), it is also clear that $v_\epsilon$ converges weakly to zero, meaning it is a Weyl sequence and so $\lambda \in \sigma _{ess}(M)$. This completes the proof. $\square$

## Full spectrum bound

In Sect. 5, we completely characterized the essential spectrum of *L*. We are unable to do the same for the full spectrum, but we can constrain $\sigma (L)$ as in [22, Theorem 1]. For completeness, we include a proof of Proposition 3.

### Proposition 3

(Dyson and Schutz) The spectrum $\sigma (L)$ satisfies

1. $$\begin{aligned} \sigma (L) \subseteq \textrm{i}\mathbb {R}\cup \{ \lambda \in \mathbb {C}\,\ |\operatorname {Im}(\lambda )| \le |\Omega | \}; \end{aligned}$$
2. while $A_2$ is bounded below by $\gamma (A_2)$, $\lambda \in \sigma (L)$ and $\lambda \notin i \mathbb {R}$,
  $$\begin{aligned} |\lambda |^2 \le \max (0,-\gamma (A_2)). \end{aligned}$$

### Proof

We begin with introducing the sets

$$\begin{aligned} \mathscr {R}_\Omega = \textrm{i}\mathbb {R} \cup \{ \lambda \in \mathbb {C}\,\ |\operatorname {Im}(\lambda )| \le \Vert R_\Omega \Vert \} \end{aligned}$$

and

$$\begin{aligned} \mathscr {S}(c) = \{ \lambda = \zeta + \textrm{i}\xi \in \mathbb {C}\,\ \zeta , \xi \in \mathbb {R},\ \zeta ^2 - \xi ^2 \le c \}. \end{aligned}$$

**Step 1**: *rough bound for* $\sigma (L)$. Let us first assume that $A_2$ is bounded below by $\gamma (A_2)$. Setting $\lambda = \zeta + i \xi$ with $\zeta$, $\xi \in \mathbb {R}$ and taking $u \in D(A_2)$, we first estimate

71 $$\begin{aligned} \Vert (\lambda ^2 \operatorname {Id}+ A_2) u \Vert _H^2 = \Vert (\zeta ^2 - \xi ^2) u + A_2 u + 2 \textrm{i}\zeta \xi u \Vert _H^2 \nonumber \\ = \Vert (\zeta ^2 - \xi ^2) u + A_2 u \Vert _H^2 + \Vert 2 \textrm{i}\zeta \xi u \Vert _H^2 \ge ((\zeta ^2 - \xi ^2 + \gamma (A_2))^2 + 4 \zeta ^2 \xi ^2) \Vert u \Vert _H^2 \end{aligned}$$

provided that $\zeta ^2 - \xi ^2 + \gamma (A_2) > 0$. Hence, in this part of the complex plane, $\lambda ^2 \operatorname {Id}+ A_2$ is invertible. We write

$$\begin{aligned} c_1 = \max (0,-\gamma (A_2)) + 1 \end{aligned}$$

and find that $(\lambda ^2 \operatorname {Id}+ A_2)^{-1}$ is a bounded operator for $\lambda \in \mathscr {S}(c_1)^c$ with

72 $$\begin{aligned} \Vert (\lambda ^2 \operatorname {Id}+ A_2)^{-1} \Vert \le (( \zeta ^2 - \xi ^2 + \gamma (A_2))^2 + 4 \zeta ^2 \xi ^2)^{-1/2}. \end{aligned}$$

Now, for $\lambda \in \mathscr {S}(c_1)^c$ we have

$$\begin{aligned} L(\lambda ) = (\lambda ^2 \operatorname {Id}+ A_2)( \operatorname {Id}+ (\lambda ^2 \operatorname {Id}+ A_2)^{-1} 2 \lambda R_\Omega ), \end{aligned}$$

and using (72)

$$\begin{aligned} \Vert (\lambda ^2 \operatorname {Id}+ A_2)^{-1} 2 \lambda R_\Omega \Vert \le 2 |\lambda | \, \Vert R_\Omega \Vert ((\zeta ^2 - \xi ^2 + \gamma (A_2))^2 + 4 \zeta ^2 \xi ^2)^{-1/2} \\ = 2 |\lambda | \, \Vert R_\Omega \Vert [|\lambda |^4 + 2 (\zeta ^2 - \xi ^2) \gamma (A_2) + \gamma (A_2)^2]^{-1/2}. \end{aligned}$$

If $|\lambda |^2 = \zeta ^2 + \xi ^2> c_2 >0$ is such that the right-hand side is less than 1/2, then

$$\begin{aligned} \operatorname {Id}+ (\lambda ^2 \operatorname {Id}+ A_2)^{-1} 2 \lambda R_\Omega \end{aligned}$$

is invertible and so

$$\begin{aligned} L(\lambda )^{-1} = (\operatorname {Id}+ (\lambda ^2 \operatorname {Id}+ A_2)^{-1} 2 \lambda R_\Omega )^{-1} (\lambda ^2 \operatorname {Id}+ A_2)^{-1} \end{aligned}$$

is bounded. Therefore, if we set $c_3 = \max (c_1,c_2)$, then $\mathscr {S}(c_3)^c \subset \rho (L)$. Consequently, we have $\sigma (L) \subset \mathscr {S}(c_3)$.

**Step 2**: *proof of Proposition* 3,* 1.* We assume that $\lambda \in \partial \sigma (L) = \sigma (L) \cap \overline{\rho (L)}$. Applying Lemma 8, we generate a sequence $\{\lambda _\ell \}_{\ell =1}^\infty \subset \rho (L)$ and displacement vectors $\{ u^\ell \}_{\ell =1}^\infty$ such that

$$\begin{aligned} \lim _{\ell \rightarrow \infty } \lambda _\ell = \lambda ,\quad \Vert u^\ell \Vert _H = 1,\quad \lim _{\ell \rightarrow \infty } \Vert L(\lambda _\ell ) u^\ell \Vert _H = 0. \end{aligned}$$

It follows that

$$\begin{aligned} \lim _{\ell \rightarrow \infty } (L(\lambda _\ell ) u^\ell ,u^\ell ) = 0. \end{aligned}$$

We define the quantities

73 $$\begin{aligned} s_\ell = \frac{1}{\textrm{i}} \, (R_\Omega u^\ell ,u^\ell ),\quad \tau _\ell = (A_2 u^\ell ,u^\ell ),\quad q_\ell = (L(\lambda _\ell ) u^\ell ,u^\ell ) \end{aligned}$$

with $s_\ell \in \mathbb {R}$, $\tau _\ell \in \mathbb {R}$ and $\lim _{\ell \rightarrow \infty } q_\ell = 0$, while

$$\begin{aligned} \lambda _\ell ^2 + 2 \textrm{i}s_\ell \lambda _\ell + \tau _\ell = q_\ell . \end{aligned}$$

Writing $\zeta _\ell = \operatorname {Re}(\lambda _\ell )$, $\xi _\ell = \operatorname {Im}(\lambda _\ell )$ and taking the imaginary part of both sides,

$$\begin{aligned} 2 \zeta _\ell \left( \xi _\ell + \frac{1}{\textrm{i}} \, (R_\Omega u^\ell ,u^\ell )\right) = \operatorname {Im}(q_\ell ). \end{aligned}$$

Because the right-hand side goes to zero as $\ell \rightarrow \infty$, we have

74 $$\begin{aligned} \lim _{\ell \rightarrow \infty } \min \left\{ |\zeta _\ell |, \left| \xi _\ell + \frac{1}{\textrm{i}} \, (R_\Omega u^\ell ,u^\ell ) \right| \right\} = 0. \end{aligned}$$

Clearly, for all $\ell$

75 $$\begin{aligned} -\Vert R_\Omega \Vert \le \frac{1}{\textrm{i}} (R_\Omega u_\ell ,u_\ell ) \le \Vert R_\Omega \Vert \end{aligned}$$

and, hence, (74) implies that

$$\begin{aligned} \lim _{\ell \rightarrow \infty } \operatorname {dist}(\lambda _\ell ,\mathscr {R}_\Omega ) = 0. \end{aligned}$$

But then $\lambda \in \mathscr {R}_\Omega$. Therefore, $\partial \sigma (L) = \sigma (L) \cap \overline{\rho (L)} \subset \mathscr {R}_\Omega$.

We will prove $\sigma (L) \subset \mathscr {R}_\Omega$ by contradiction. Assume that $\sigma (L) \not \subset \mathscr {R}_\Omega$, then there exists a $\xi _0 \in \mathbb {R}$ such that the line $M(\xi _0) = \{\zeta + \textrm{i}\xi _0 \in \mathbb {C}\ :\ \zeta \in \mathbb {R}\}$ parallel to the real axis intersects $\sigma (L) \setminus \mathscr {R}_\Omega$. From the characterization of $\mathscr {R}_\Omega$ it follows that $|\xi _0| > \Vert R_\Omega \Vert$ and that $M(\xi _0) \cap \rho (L) \ne \emptyset$ using the rough estimate $\sigma (L) \subset \mathscr {S}(c_3)$. We define the set

$$\begin{aligned} \mathscr {T}(\xi _0) = \{\zeta \in \mathbb {R}\,\ \zeta + \textrm{i}\xi _0 \in \sigma (L)\}. \end{aligned}$$

This set is a bounded and closed set in $\mathbb {R}$. We define $\zeta _1 = \max \, \mathscr {T}(\xi _0)$, $\zeta _2 = \min \, \mathscr {T}(\xi _0)$. Because $\sigma (L)$ is a closed set, $\lambda _1 = \zeta _1 + \textrm{i}\xi _0$ and $\lambda _2 = \zeta _2 + \textrm{i}\xi _0$ belong to $\sigma (L)$; from the definition of $\zeta _1$, $\zeta _2$ it follows that $\lambda _1$ and $\lambda _2$ belong to $\partial \sigma$. However, we proved that $\partial \sigma \subset \mathscr {R}_\Omega$ and that $|\xi _0| > \Vert R_\Omega \Vert$ and, hence, $\lambda _1 = \lambda _2 = \textrm{i}\xi _0$. This is a contradiction which completes the proof of part *1* of Proposition 3.

**Step 3**: *proof of Proposition* 3,* 2.* We assume that $\lambda \in \partial \sigma (L) = \sigma (L) \cap \overline{\rho (L)}$ with $\operatorname {Re}(\lambda ) \ne 0$. Applying Lemma 8, we generate a sequence $\{\lambda _\ell \}_{\ell =1}^\infty \subset \rho (L)$ and displacement vectors $\{u^\ell \}_{\ell =1}^\infty$ such that

$$\begin{aligned} \lim _{\ell \rightarrow \infty } \lambda _\ell = \lambda ,\quad \Vert u^\ell \Vert = 1,\quad \lim _{\ell \rightarrow \infty } \Vert L(\lambda _\ell ) u^\ell \Vert = 0 \end{aligned}$$

as before. Also, let $s_\ell$, $\tau _\ell$ and $q_\ell$ be as in (73). By (75), $s_\ell$ is bounded and since $q_\ell \rightarrow 0$ and $\lambda _\ell \rightarrow \lambda$, $\tau _\ell$ must also be bounded. Therefore $s_\ell$ and $\tau _\ell$ have convergent subsequences and, by passing to a subsequence if necessary, we can assume without loss of generality that $s_\ell$ and $\tau _\ell$ converge, respectively, to some *s* and $\tau \in \mathbb {R}$. Then, taking the limit in (73) we obtain

$$\begin{aligned} \lambda ^2 + 2 i s \lambda + \tau = 0, \end{aligned}$$

which implies

$$\begin{aligned} \lambda = -is \pm \sqrt{-s^2 - \tau }. \end{aligned}$$

If $-s^2 - \tau \le 0$, then $\lambda \in i \mathbb {R}$ which we have excluded by assumption. Therefore, $s^2 + \tau < 0$ and

$$\begin{aligned} |\lambda |^2 = s^2 + (-s^2 - \tau ) = -\tau \le -\gamma (A_2) \end{aligned}$$

This proves that if $\lambda \in \partial \sigma (L) {\setminus } \textrm{i}\mathbb {R}$, then $|\lambda |^2 \le \max (0,-\gamma (A_2))$.

Next, we prove that $\lambda \in \sigma (L) \setminus \textrm{i}\mathbb {R}$ implies that $|\lambda |^2 \le \max (0,-\gamma (A_2))$. We introduce

$$\begin{aligned} \mathscr {R}_\Omega ^\prime = \{\lambda \in \mathbb {C}\,\ |\operatorname {Im}\lambda | \le \Vert R_\Omega \Vert ,\ |\lambda |^2\le \max (0,-\gamma (A_2))\}. \end{aligned}$$

We already know that $\partial \sigma (L) {\setminus } \textrm{i}\mathbb {R} \subset \mathscr {R}_\Omega ^\prime$. Now, we assume that

76 $$\begin{aligned} (\sigma (L) \setminus \textrm{i}\mathbb {R}) \not \subset \mathscr {R}_\Omega ^\prime , \end{aligned}$$

and let us use the same notation $M(\xi _0)$ and $\mathscr {T}(\xi _0)$ as in *Step 2* above. Then there exists a $\xi _0 \in [-\Vert R_\Omega \Vert ,\Vert R_\Omega \Vert ]$ such that the line $M(\xi _0)$ parallel to the real axis intersects $(\sigma (L) {\setminus } \textrm{i}\mathbb {R}) {\setminus } \mathscr {R}_\Omega ^\prime$. With this $\xi _0$, $\mathscr {T}(\xi _0)$ is a bounded and closed set using the result obtained in *Step 1*. We define, again, $\zeta _1 = \max \, \mathscr {T}(\xi _0)$, $\zeta _2 = \min \, \mathscr {T}(\xi _0)$. Then $\lambda _1 = \zeta _1 + \textrm{i}\xi _0$, $\lambda _2 = \zeta _2 + \textrm{i}\xi _0$ necessarily belong to $\partial \sigma (L)$. Due to assumption (76), we have $|\lambda _1| > \max (0,-\gamma (A_2)$ or $|\lambda _2| > \max (0,-\gamma (A_2))$. This is a contradiction to $\partial \sigma (L) \subset \mathscr {R}_\Omega ^\prime$. $\square$

The next lemma was used in the proof of Proposition 3.

### Lemma 8

Suppose that $\lambda \in \partial \sigma (L)$. Then there exists a sequence $\lambda _\ell \in \rho (L)$ and $u^\ell \in D(L)$ such that

$$\begin{aligned} \lim _{\ell \rightarrow \infty } \lambda _\ell = \lambda ,\quad \Vert u^\ell \Vert _H = 1,\quad \lim _{\ell \rightarrow \infty } \Vert L(\lambda _\ell ) u^\ell \Vert _H = 0. \end{aligned}$$

### Proof

Let $\lambda \in \partial \sigma (L)$ and so there exists a sequence $\lambda _\ell \in \rho (L)$ such that $\lambda _\ell \rightarrow \lambda$. Since $\sigma (L)$ is closed, $\lambda \in \sigma (L)$. Suppose that $L(\lambda )$ is not injective. Then, the lemma is proven by taking $u_\ell$ a constant sequence with norm 1 in the kernel of $L(\lambda )$. So, assume now that $L(\lambda )$ is injective. Then $L^{-1}(\lambda )$ is defined on some domain in *H*, and if this domain is all of *H* then $L(\lambda )^{-1}$ must be continuous. Thus, there must exist $f \in H$ which is not in the range of $L(\lambda )$. Define

$$\begin{aligned} v_\ell = L(\lambda _\ell )^{-1} f. \end{aligned}$$

We then claim that some subsequence of $u_\ell = v_\ell /\Vert v_\ell \Vert _H$ is a sequence of unit vectors that satisfies

77 $$\begin{aligned} \lim _{\ell \rightarrow \infty }\Vert L(\lambda _\ell ) u^\ell \Vert _H = 0. \end{aligned}$$

We will argue by contradiction.

Indeed, suppose that no subsequence of $u_\ell$ as defined above satisfies (77). Then

$$\begin{aligned} \Vert L(\lambda _\ell ) u^\ell \Vert _{H} \ge C > 0 \Rightarrow \Vert f\Vert _H = \Vert L(\lambda _\ell ) v^\ell \Vert _H \ge C \Vert v^\ell \Vert _{H} \end{aligned}$$

for some constant *C* and all $\ell$. Therefore, since $v_\ell$ is bounded, it must have a weakly convergent subsequence: say $v_\ell$ converges to *v* weakly.

Now suppose $g \in D(A_2)$. Then

$$\begin{aligned} \langle f, g \rangle _H = \langle L(\lambda _\ell ) v^\ell , g \rangle _H = \langle v^\ell , L(\lambda _\ell )^* g \rangle _H = \langle v^\ell , (L(\lambda _\ell )^* - L(\lambda )^*) g \rangle _H + \langle v^\ell , L(\lambda )^*g \rangle _H \end{aligned}$$

Taking the limit as $\ell \rightarrow \infty$ gives

$$\begin{aligned} \langle f, g \rangle _H = \langle v, L(\lambda )^*g \rangle _H. \end{aligned}$$

Therefore $v \in D(L)$ and $L(\lambda )v = f$. This is a contradiction since *f* was assumed to be outside the range of *L*. This completes the proof. $\square$

If $\gamma (A_2) \ge 0$, it follows immediately from Proposition 3 that $\sigma \subseteq \textrm{i}\mathbb {R}$, but this is unlikely to be the case. In general, Proposition 3 provides an upper bound on the full spectrum $\sigma (L)$ which is illustrated in Fig. 2.

## Discussion

We precisely characterized the spectrum of rotating truncated gas planets for both variable densities and variable positive and negative squared Brunt–Väisälä frequencies. Acoustic modes correspond with part of the point spectrum (while other modes such as quasi-rigid body modes are also associated with the point spectrum) and inertia–gravity modes with the essential spectrum. We presented a partial resolution of the identity with acoustic modes which reveals inaccuracies in common approaches to compute these.

A further study of the dynamics and attractors associated with the inertia–gravity modes described in this paper will be left for future research. We note that such analysis was carried out by Colin de Verdière and Vidal [13]. In preparation for this, making the connection to their work explicit, we briefly relate our formulation to theirs. We introduced

78 $$\begin{aligned} \tilde{s} = \nabla \rho _0 - \frac{\rho _0}{c^2} g_0' \end{aligned}$$

and identified the dynamic pressure as

79 $$\begin{aligned} P = -c^2 [\nabla \cdot (\rho _0 u) - \tilde{s} \cdot u] \end{aligned}$$

or

80 $$\begin{aligned} P = -\rho _0\ [c^2 \nabla \cdot u + g_0' \cdot u]. \end{aligned}$$

Using that

81 $$\begin{aligned} \tilde{s} \cdot u = \frac{\tilde{s} \cdot g_0'}{|g_0'|^2} (g_0' \cdot u) = \frac{N^2}{|g_0'|^2} (g_0' \cdot (\rho _0 u)) \end{aligned}$$

as $\nabla \rho _0$ and $g_0'$ must be parallel, we obtain

82 $$\begin{aligned} P = -c^2 \left[ \nabla \cdot (\rho _0 u) - \frac{N^2}{|g_0'|^2} (g_0' \cdot (\rho _0 u)) \right] . \end{aligned}$$

While introducing the particle velocity, $v = \partial _t u$, Eqs. (10) and (2) are equivalent to the system

83 $$\begin{aligned} \partial _t \rho + \nabla \cdot (\rho _0 v)= &   0, \end{aligned}$$

84 $$\begin{aligned} \partial _t (\rho _0 v) + 2 \Omega \times (\rho _0 v)= &   -\nabla P + \rho g_0' - \rho _0 \nabla \Phi ', \end{aligned}$$

85 $$\begin{aligned} \partial _t P= &   c^2 \left[ \partial _t \rho + \frac{N^2}{|g_0'|^2} (g_0' \cdot (\rho _0 v)) \right] , \end{aligned}$$

supplemented with (2), which is equivalent to the system in linearized hydrodynamics as in [39]

86 $$\begin{aligned} \partial _t \rho + \nabla \cdot (\rho _0 v)= &   0, \end{aligned}$$

87 $$\begin{aligned} \partial _t (\rho _0 v) + 2 \Omega \times (\rho _0 v)= &   -\nabla P + \rho g_0' - \rho _0 \nabla \Phi ', \end{aligned}$$

88 $$\begin{aligned} \partial _t P + v \cdot \nabla P_0= &   c^2 [ \partial _t \rho + v \cdot \nabla \rho _0 ] \end{aligned}$$

as $\nabla P_0 = -\rho _0 g_0'$. In the Cowling approximation, one drops the term $-\rho _0 \nabla \Phi '$. If $u \in \ker (T)$, then $P = 0$ and

89 $$\begin{aligned} \rho g_0' = -(\nabla \cdot (\rho _0 u)) g_0' = -(\tilde{s} \cdot u) g_0' = -N^2 \hat{g}_0' (\hat{g}_0' \cdot \rho _0 u). \end{aligned}$$

Then, (84,85) is seen to be equivalent to

$$\begin{aligned} \partial _t v + 2 \Omega \times v + N^2 \hat{g}_0' (\hat{g}_0' \cdot u) = 0, \quad Tu = 0. \end{aligned}$$

which is closely related to (41). Upon first introducing

90 $$\begin{aligned} \rho ' = N (\underbrace{\hat{g}_0' \cdot u}_{u_{\parallel }}), \end{aligned}$$

this equation can be written as the system

91 $$\begin{aligned} (\partial _t + A) \left( \begin{array}{c} v\\ \rho ' \end{array}\right) = 0\quad \text {with}\ A = \left( \begin{array}{cc} 2 \Omega \times &  N \hat{g}_0' \\ -N \hat{g}_0'^T &  0 \end{array}\right) ,\quad T v = 0. \end{aligned}$$

In [13], this system is formed by expressing *v* in an orthogonal basis where one of the basis vectors is $\hat{g}_0'$. Including the projectors,

92 $$\begin{aligned} \pi _2' \left( \begin{array}{c} v \\ \rho ' \end{array}\right) = \left( \begin{array}{c} \pi _2 v \\ \rho ' \end{array}\right) , \end{aligned}$$

the system takes the form

93 $$\begin{aligned} (\partial _t + H) \left( \begin{array}{c} v\\ \rho ' \end{array}\right) = 0\quad \text {with}\ H = \pi _2' A \pi _2' \end{aligned}$$

as in Colin de Verdière and Vidal [13], who considered the case when $\hat{g}_0'$ and *N* are constants. (These authors consider the further spectral analysis of this equation which is, in turn related to the work of [20] in case the (compact) manifold would not have a boundary.) The system needs to be supplemented with the boundary condition $u\cdot n|_{\partial M} = 0$ (see (20)). *H* is identified with the Poincaré operator. The spectrum of *H* is $\sigma _{ess}(L_{22})$.

We can write the constrained system (91) (in the Cowling approximation) in the form

94 $$\begin{aligned} \left( \begin{array}{cc} -\operatorname {i} \lambda + \pi '_2 A &  \begin{array}{c} \nabla \\ 0 \end{array} \\ \begin{array}{cc} T &  0 \end{array}&0 \end{array}\right) \left( \begin{array}{c} v \\ \rho ' \\ P \end{array}\right) = 0. \end{aligned}$$

The principal, $\sigma _p(\pi _2)$, corresponds with the Leray projector and is given by (22). Consistent with the Leray projector, we may restrict $u \in \ker T_1$, $T_1 u = \rho _0 \nabla \cdot u$ when $P = P_1 = \rho _0 g_0' \cdot u$ and is nonvanishing. Then

95 $$\begin{aligned} \left( \begin{array}{cc} -\operatorname {i} \lambda + \sigma _p(\pi _2') A &  \begin{array}{c} \nabla \\ 0 \end{array} \\ \begin{array}{cc} \nabla \cdot &  0 \end{array}&0 \end{array}\right) \left( \begin{array}{c} v \\ \rho ' \\ P_1 \end{array}\right) = 0. \end{aligned}$$

Keeping the principal parts, eliminating *v* and $\rho '$, leads to a Poincaré equation for $P_1$, $S_{\omega } P_1 = 0$, where the principal symbol of $S_{\omega }$ is given by

$$\begin{aligned} \begin{aligned} s_{\omega }(x,\xi )&= \det \left( \begin{array}{cc} -\operatorname {i} \lambda + \sigma _p(\pi _2')(\xi ) A(x) &  \begin{array}{c} \operatorname {i} \xi \\ 0 \end{array} \\ \begin{array}{cc} \operatorname {i} \xi ^T &  0 \end{array}&0 \end{array}\right) \\&= -i \lambda |\xi |^2 (\lambda ^2 + 4 \Omega _\xi ^2 + N^2 |P_{\xi }^{\perp } \hat{g}_0'|^2). \end{aligned} \end{aligned}$$

This determinant can be calculated by considering the matrix in an orthonormal basis that includes $(\xi /|\xi |,0,0)^T)$ as one of the basis vectors. Therefore, $S_\omega$ is elliptic except when $\lambda = 0$ or

$$\begin{aligned} \lambda ^2 = - 2 \Omega _\xi ^2 - N^2 |P_\xi ^\perp \hat{g}_0'|^2, \end{aligned}$$

which corresponds with Lemma 5.

Future work includes a generalization to the precise characterization of the spectra of rotating terrestrial planets involving boundary conditions at the core–mantle interface different from the ones appearing in the present results, and extending the work of Valette [49]. It also includes removing the truncation employed in the present analysis of gas planets by letting $c^2$ vanish (proportional to the pseudo-enthalpy in a polytropic model) and $N^2$ blow-up (proportional to $c^{-2}$ in a polytropic model) at the boundary, see Prat et al. [39].

## Appendix A. A study of $\operatorname {Ker}(A_2)$

In this appendix, we study $\operatorname {Ker}(A_2)$ which leads to the introduction of rigid body motions with corresponding eigenvalues not contained in $\sigma _1$. Here we review modes associated with these motions and provide explicit calculations.

Quasi-rigid displacements are those with zero strain. The vanishing strain condition implies that they must be of the form

96 $$\begin{aligned} u(x) = t + k \wedge x,\quad t, k \in \mathbb {C}^3. \end{aligned}$$

We briefly demonstrate that such quasi-rigid displacements are zero eigenfunctions in the case of a non-rotating planet. In a rotating planet, the quasi-rigid motions are still eigenfunctions, but they have different eigenvalues as we will demonstrate below.

### A.1. Non-rotating planet

To consider the non-rotating case, we first calculate

$$\begin{aligned} u(x) \cdot \nabla \nabla \Phi ^0(x) = G \lim _{\epsilon \rightarrow 0^+} \int \limits _{M \setminus B_\epsilon (x)} \frac{x - x'}{|x - x'|^3}\cdot u(x)\ \nabla \rho ^0 (x')\ \textrm{d}x', \\ \nabla S(u)(x) = -G \lim _{\epsilon \rightarrow 0^+} \int \limits _{M \setminus B_\epsilon (x)} \nabla u(x') \frac{x - x'}{|x - x'|^3}\ \rho ^0(x') + \frac{x - x'}{|x - x'|^3}\cdot u(x')\ \nabla \rho ^0(x')\ \textrm{d}x', \end{aligned}$$

and

$$\begin{aligned} \nabla \Phi ^0(x)^T \nabla u(x) = G \lim _{\epsilon \rightarrow 0^+} \int \limits _{M \setminus B_\epsilon (x)} \frac{(x - x')^T}{|x - x'|^3} \nabla u(x)\ \rho ^0(x') \textrm{d}x'. \end{aligned}$$

Using these formulae and the facts that *u* given by (96) has zero strain, constant antisymmetric first differential (that is, $\nabla u$ is a constant antisymmetric matrix), and vanishing second derivatives we find that for such *u*

$$\begin{aligned} \begin{aligned} A_2^0 u(x)&= \nabla S(u)(x) - \nabla \Phi ^0(x)^T \nabla u(x) + u \cdot \nabla \nabla \Phi ^0(x) \\&= G \lim _{\epsilon \rightarrow 0^+} \int \limits _{M \setminus B_\epsilon (x)} \frac{(x - x')^T}{|x - x'|^3} (\nabla u(x') - \nabla u(x))\ \rho ^0(x') \\&\hspace{108.405pt}+ \frac{x - x'}{|x - x'|^3} \cdot (u(x) - u(x'))\ \nabla \rho ^0(x') \textrm{d}x' \\&= G \lim _{\epsilon \rightarrow 0^+} \int \limits _{M \setminus B_\epsilon (x)} \frac{x - x'}{|x - x'|^3} \cdot (k \wedge (x - x'))\ \nabla \rho ^0(x') \textrm{d}x' \\&= 0 \end{aligned} \end{aligned}$$

This calculation shows that, as stated above, in the non-rotating case the quasi-rigid motions are eigenfunctions associated with the zero eigenvalue.

### A.2. Rotating planet

Now we consider the rotating case. First, from the above calculation, since $A_2^0 u = 0$ for the quasi-rigid motions, we find

$$\begin{aligned} \begin{aligned} A_2 u(x)&= u(x) \cdot \nabla \nabla \Psi ^s(x) - \nabla \Psi ^s(x)^T \nabla u(x) \\&= (\Omega \cdot u)\ \Omega - \Omega ^2\ u + (\Omega \cdot x)\ \Omega ^T \nabla u - \Omega ^2\ x^T\nabla u \\&= (\Omega \cdot t)\ \Omega + \Omega \cdot (k \wedge x)\ \Omega - \Omega ^2\ t - \Omega ^2 k \wedge x - (\Omega \cdot x)\ k \wedge \Omega + \Omega ^2 \ k \wedge x \\&= (\Omega \cdot t)\ \Omega + \Omega \cdot (k \wedge x)\ \Omega - \Omega ^2\ t - (\Omega \cdot x)\ k \wedge \Omega . \end{aligned} \end{aligned}$$

From this formula we can immediately see that when $t = c_t \Omega$ and $k = c_k \Omega$,

$$\begin{aligned} A_2 u = c_t\ \Omega ^2\ \Omega - c_t\ \Omega ^2 \ \Omega = 0. \end{aligned}$$

Thus the quasi-rigid motion

$$\begin{aligned} u(x) = c_t\ \Omega + c_k\ \Omega \wedge x, \end{aligned}$$

is an eigenfunction associated with zero eigenvalue in the rotating case. The first term on the right-hand side is identified with the *axial translational mode*, while the second term on the right-hand side is identified with the *axial spin mode*.

The other rigid motions correspond with nonzero eigenvalues. We must also include the first and second-order terms. Thus, we calculate for quasi-rigid motions:

$$\begin{aligned} \begin{aligned} L(\lambda ) u&= (\lambda ^2 \operatorname {Id}+ 2 \lambda R_{\Omega } + A_2) u \\&= \lambda ^2 t + \lambda ^2 k \wedge x + 2 \lambda \Omega \wedge t + 2 \lambda \Omega \wedge (k \wedge x) + (\Omega \cdot t) \ \Omega + \Omega \cdot (k \wedge x)\ \Omega - \Omega ^2\ t - (\Omega \cdot x)\ k \wedge \Omega . \end{aligned} \end{aligned}$$

First, let us consider the case when $t \perp \Omega$ and $k = 0$. Then we have

$$\begin{aligned} L(\lambda )u = (\lambda ^2 - \Omega ^2) t + 2 \lambda \Omega \wedge t. \end{aligned}$$

Choosing any $a \perp \Omega$ and setting

$$\begin{aligned} t_\pm = a \pm \textrm{i}\frac{\Omega \wedge a}{|\Omega |} m \end{aligned}$$

we have

$$\begin{aligned} \Omega \wedge t_\pm = \mp \textrm{i}| \Omega | t_\pm . \end{aligned}$$

Using this we see that with $\lambda _\pm = \pm \textrm{i}|\Omega |$

$$\begin{aligned} \begin{aligned} L(\lambda _\pm )t_\pm&= - 2 \Omega ^2 t_\pm + 2 (\pm \textrm{i}|\Omega |) (\mp \textrm{i}| \Omega | t_\pm ). \end{aligned} \end{aligned}$$

Therefore, we have a two-dimensional space of eigenfunction and eigenvalue pairs given by

$$\begin{aligned} \lambda _\pm = \pm \textrm{i}|\Omega |, \quad t_\pm = a \pm \textrm{i}\frac{\Omega \wedge a}{|\Omega |}, \quad k = 0. \end{aligned}$$

These are the so-called *equatorial translation modes*. We note that the translation modes are not contained in *H*; thus the only mode in $\operatorname {Ker} A_2$ playing a role is the axial spin mode.

Now let us consider the case $t = 0$, and $k \perp \Omega$. Choosing any $a \perp \Omega$ we have as before

$$\begin{aligned} k_\pm = a \pm \textrm{i}\frac{\Omega \wedge a}{|\Omega |} \Rightarrow \Omega \wedge k_\pm = \mp \textrm{i}| \Omega | k_\pm . \end{aligned}$$

Again, taking $\lambda _\pm = \pm \textrm{i}|\Omega |$, we find that

$$\begin{aligned} \begin{aligned} L(\lambda _\pm ) k_\pm \wedge x&= - \Omega ^2 k_\pm \wedge x + 2 (\pm \textrm{i}|\Omega |) (\Omega \cdot x) k_\pm + \Omega \cdot \left( k_\pm \wedge x \right) \Omega - (\Omega \cdot x) \left( k_\pm \wedge \Omega \right) \\&= - \Omega ^2 k_\pm \wedge x \pm 2 \textrm{i}|\Omega | (\Omega \cdot x) k_\pm + \Omega ^2 k_\pm \wedge x + 2 (x \cdot \Omega ) \Omega \wedge k_{\pm } \\&= 0. \end{aligned} \end{aligned}$$

Therefore, there is also a two-dimensional space of eigenfunction and eigenvalue pairs given by

$$\begin{aligned} \lambda _\pm = \pm \textrm{i}|\Omega |, \quad t = 0,\quad k_\pm = a \pm \textrm{i}\frac{\Omega \wedge a}{|\Omega |}. \end{aligned}$$

These are the so-called tilt-over modes. We note that tilt-over modes are well-known (according to Chandrasekhar [8]) transversely skewed oscillations that can occur in rotating, precessing bodies.

These calculations show that in moving from the non-rotating model to the rotating one the six-dimensional eigenspace of quasi-rigid modes with eigenvalue zero is split into three separate two-dimensional eigenspaces with eigenvalues $\pm \textrm{i}|\Omega |$ and 0.

## Footnotes

1 This form follows from the incremental Lagrangian stress formulation in the acoustic limit as presented, for example, in [17].

2 That is, a local approximation assuming $g_0' \cdot \Omega$ is constant.

3 A closed linear operator between Hilbert spaces is called Fredholm if its nullspace is finite dimensional and its range has finite codimension. Thus, at points in the essential spectrum there may be infinitely many linearly independent eigenfunctions.
